# Total biosynthesis of opiates by stepwise fermentation using engineered *Escherichia coli*

**DOI:** 10.1038/ncomms10390

**Published:** 2016-02-05

**Authors:** Akira Nakagawa, Eitaro Matsumura, Takashi Koyanagi, Takane Katayama, Noriaki Kawano, Kayo Yoshimatsu, Kenji Yamamoto, Hidehiko Kumagai, Fumihiko Sato, Hiromichi Minami

**Affiliations:** 1 Applied Microbiology laboratory, Research Institute for Bioresources and Biotechnology, Ishikawa Prefectural University, 1-308 Suematsu, Ishikawa 921-8836, Japan; 2 Department of Applied Molecular Biology, Graduate School of Biostudies, Kyoto University, Oiwake-cho, Kitashirakawa, Kyoto 606-8502, Japan; 3 Breeding and Physiology Laboratory, Tsukuba Research Center for Medicinal Plant Resources, National Institutes of Biomedical Innovation, Health and Nutrition, 1-2 Hachimandai, Tsukuba 305-0843, Japan; 4 Department of Plant Gene and Totipotency, Division of Integrated Life Science, Graduate School of Biostudies, Kyoto University, Oiwake-cho, Kitashirakawa, Kyoto 606-8502, Japan

## Abstract

Opiates such as morphine and codeine are mainly obtained by extraction from opium poppies. Fermentative opiate production in microbes has also been investigated, and complete biosynthesis of opiates from a simple carbon source has recently been accomplished in yeast. Here we demonstrate that *Escherichia coli* serves as an efficient, robust and flexible platform for total opiate synthesis. Thebaine, the most important raw material in opioid preparations, is produced by stepwise culture of four engineered strains at yields of 2.1 mg l^−1^ from glycerol, corresponding to a 300-fold increase from recently developed yeast systems. This improvement is presumably due to strong activity of enzymes related to thebaine synthesis from (*R*)-reticuline in *E. coli*. Furthermore, by adding two genes to the thebaine production system, we demonstrate the biosynthesis of hydrocodone, a clinically important opioid. Improvements in opiate production in this *E. coli* system represent a major step towards the development of alternative opiate production systems.

## Abstract (web summary)

Opiates—the gold standard for pain relief—are currently produced by extraction from opium poppies. Here the authors show that bacteria can serve as an efficient and flexible platform for the production of opiates by demonstrating the total synthesis of Thebaine and hydrocodone from stepwise fermentation in *E. coli*.

## Graphical abstract

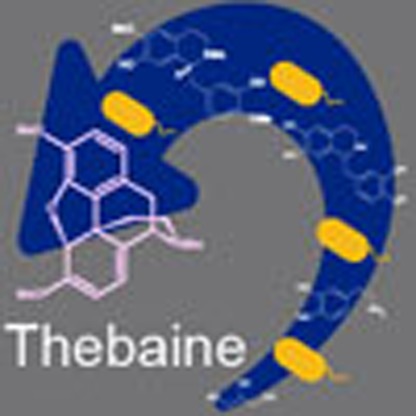

Opiates are commonly used painkillers that are prescribed for illnesses ranging from cancer to rheumatism and toothaches. They are typically extracted from plants, and more efficient production of opiate has been attempted. Biotechnology methods for poppy cultivation to increase yields have been studied extensively^1^,^2^,^3^; however, complex and unknown mechanisms that regulate biosynthetic pathways might make it difficult to increase opiate yields. Although there are several examples of successful chemical opiate synthesis^4^, cost-effective methods have not been established because of the complex molecular structure of opiates. Alternatively, as a next-generation strategy, complete biosynthesis of opiates via microbes has also attracted attention because it does not require specific substrates other than inexpensive carbon sources (for example, glucose or glycerol)^5^,^6^,^7^,^8^, and has potential for improvements in quality and quantity.

The complete biosynthesis of the opiate thebaine and opioid hydrocodone in yeast are the first examples of successful production from simple substrates^8^. The yeast fermentation system is sophisticated; however, thebaine and hydrocodone yields are still limited in that system.

Material production using engineered *E. coli* has been studied extensively since the 1960s^9^, and information related to metabolic engineering has accumulated. The major production targets are primary metabolites, including amino acids, which have yields of ∼1 mol l^−1^. Because opiates are synthesized from two L-tyrosine molecules, high amino-acid productivity by *E. coli* would be helpful to construct a practical production system for opiates. *E. coli* exhibits higher productivity of opiate intermediates, such as L-tyrosine^10^, L-dopa^5^,^11^, tetrahydropapaveroline (THP)^12^ and reticuline^12^, than yeast systems^6^,^7^,^13^,^14^. Therefore, to construct a practical opiate production system, we chose *E. coli* as a production host.

Here we demonstrate the total synthesis of opiates using four *E. coli* strains.

## Results

### (*R*)-reticuline production by methyltransferases

**Figure 1:**
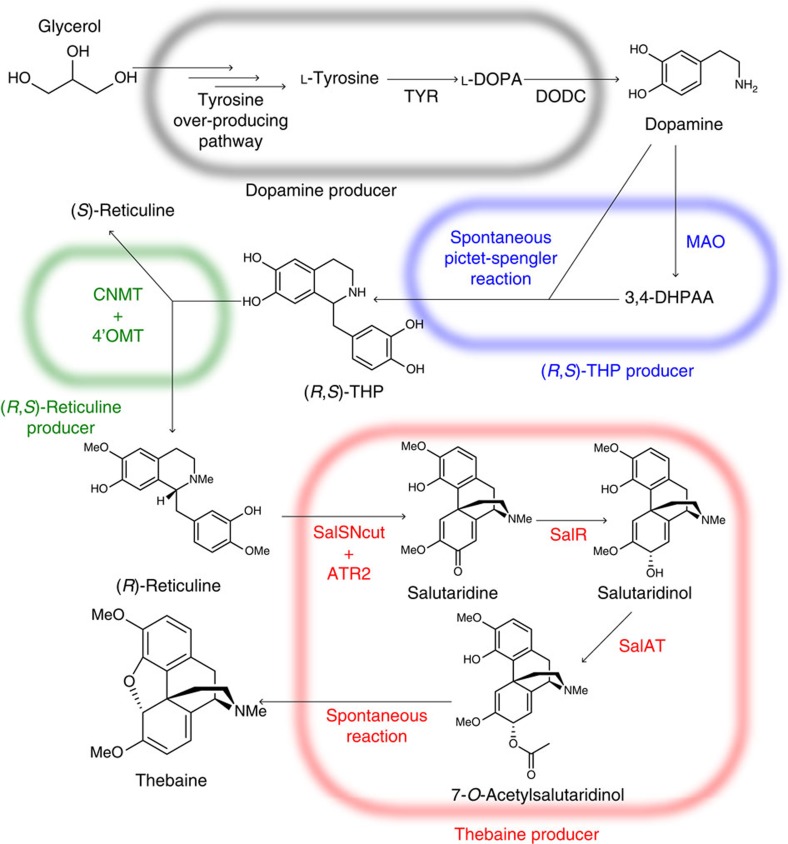
Total biosynthesis of thebaine using four-step culture. The brackets indicate the reactions in the individual strain of each culture step. ATR2, NADPH-cytochrome P450 reductase 2; CNMT, coclaurine *N*-methyltransferase; DODC, dopa decarboxylase; MAO, monoamine oxidase; SalSNcut, N-terminally-truncated salutaridine synthase; SalR, salutaridine reductase; SalAT, salutaridinol acetyltransferase; TYR, tyrosinase; 3,4-DHPAA, 3,4-dihydroxyphenylacetaldehyde; 4′OMT, 3′-hydroxy-*N*-methylcoclaurine 4′-*O*-methyltransferase.

**Figure 2:**
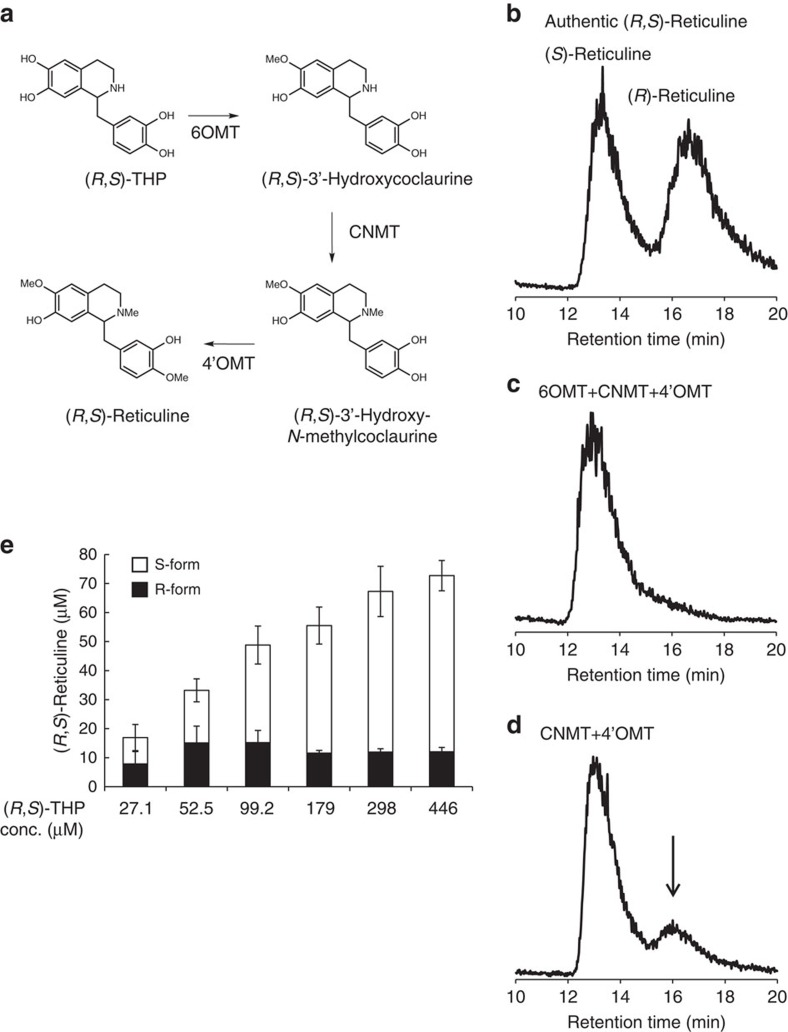
Total biosynthesis of (*R*,*S*)-reticuline. (**a**) (*R*,*S*)-Reticuline synthetic pathway from (*R*,*S*)-THP. (**b**) The chirality analysis of pure (*R*,*S*)-reticuline. (**c**) The products from the culture of AN1752. (**d**) The products from the culture of AN1600. Experiments in **b**,**c** were conducted three times, and same tendency was observed. (**e**) (*R*,*S*)-Reticuline production following the addition of various concentrations of (*R*,*S*)-THP to the AN1600 culture. The error bar indicates the standard deviation of three independent experiments.

Previously, we achieved the total biosynthesis of (*S*)-reticuline, a precursor of thebaine, from glycerol in a shake-flask culture (yield, 103 μM) using an engineered *E. coli* strain^15^. Previous studies have shown that the synthesis of the R-form of reticuline should be the initial production step (Fig. 1 and Supplementary Fig. 1)^5^. To produce (*R*)-reticuline, we examined the combination of three methyltransferases derived from *Coptis japonica*: 6-*O*-methyltransferase (6OMT)^16^, coclaurine *N*-methyltransferase (CNMT)^17^ and 4′-*O*-methyltransferase (4′OMT)^18^; these would theoretically synthesize racemic forms of reticuline from (*R*,*S*)-THP (Fig. 2a). However, only the *S*-form is produced by the strain that expresses the three methyltransferases (AN1752) cultured in (*R*,*S*)-THP-supplemented medium (Fig. 2b,c). After extensive empirical trials, we found that the CNMT- and 4′OMT-expressing strain (AN1600) could produce *R*-form reticuline (Fig. 2d). Because it has been reported that 4′OMT has 6OMT activity towards norcoclaurine^3^, we speculated that 4′OMT has 6OMT activity towards THP as well as norcoclaurine. To verify this, an enzyme assay was conducted with crude extract from 6OMT- or 4′OMT-expressing strains (AN1472 or AN1028, respectively). When 4′OMT was incubated with THP, both 6-*O*-methyl THP and 4′-*O*-methyl THP were synthesized, whereas only 6-*O*-methyl THP was detected in the reaction containing the 6OMT crude extract (Supplementary Fig. 2a,b). Indeed, 6,4′-*O*-dimethyl THP was synthesized by 4′OMT only (Supplementary Fig. 2c). These results suggested that 4′OMT has 6OMT activity towards THP, and consequently, reticuline could be produced without 6OMT (Fig. 2d). However, it was not clear why the *R*-form of reticuline was produced by the CNMT and 4′OMT expression strains, and further investigations are required to resolve this issue (described in the Supplementary Note 1).

When (*R*,*S*)-reticuline was produced from various concentrations of (*R*,*S*)-THP by the two methyltransferases expression strain (AN1600), (*S*)-reticuline production increased as (*R*,*S*)-THP increased, whereas (*R*)-reticuline formation decreased slightly, presumably owing to an S-form preference by CNMT and/or 4′OMT (Fig. 2e). The maximum amount of (*R*)-reticuline was obtained using fermentatively produced (*R*,*S*)-THP at 99.2 μM in the AN1600 culture. The (*R*,*S*)-reticuline yield reached 48±11 μM (16±3.6 mg l^−1^), including 15±4.2 μM (4.9±1.4 mg l^−1^) of the R-form. Thus, (*R*,*S*)-THP was used at ∼100 μM to produce downstream compounds in subsequent experiments.

### Selection of a suitable cytochrome P450 reductase (CPR)

During the establishment of the conditions necessary to produce (*R*,*S*)-reticuline using *E. coli* with 4′OMT and CNMT expression, the enzyme that converts (*S*)-reticuline to (*R*)-reticuline, known as STORR, was identified (Supplementary Fig. 1)^8^,^19^,^20^. Therefore, we also examined the efficiency of STORR in *E. coli*. As STORR contains a P450 enzymatic domain (CYP80Y2), and requires the reductase partner of P450 enzymes, CPR, we searched for a suitable CPR for P450s in *E. coli*. We had CPRs of *Arabidopsis thaliana* (ATR2), *Papaver somniferum* (PsCPR) and *Rattus norvegicus* (RnCPR) as laboratory stocks. Because the N-terminal deletion mutant of ATR2 is functional in *E. coli*^21^, a 45-amino-acid deletion of ATR2 (ATR2Ncut) was also prepared. The CPR activity towards bovine cytochrome *c* was verified using the crude extract from CPR-expressing strains^22^. The crude extract contained endogenous *E. coli* enzymes that catalyse nicotinamide adenine dinucleotide phosphate (NADPH); therefore, NADPHase activity was observed, even in the control sample (Supplementary Table 1). Although the activity of PsCPR was almost identical to that of the control, others had significant activity. Because ATR2 activity was strongest, it was used as a reductase partner of P450 enzymes in opiate production by *E. coli*.

### Functional expression of STORR in *E. coli*

Generally, P450 expression is difficult in *E. coli*. Because some P450 enzymes are successfully expressed in *E. coli* only after the deletion of their N-terminus^23^,^24^,^25^,^26^, N-terminal-deleted STORR (STORRNcut) was constructed in addition to full-length STORR. These STORRs were expressed with ATR2 and cultured in medium containing (*S*)-reticuline, which was biosynthesized from glycerol as described previously^15^. SDS–polyacrylamide gel electrophoresis (SDS–PAGE) analysis showed that the deletion of N-terminus was effective for protein expression (Supplementary Fig. 3a). However, neither STORR expression strain produced detectable *R*-form reticuline (Supplementary Fig. 3b–d). Because P450 is a haemoprotein, the addition of 5-aminolevulinic acid (5-ALA), a precursor of haeme, is sometimes required for functional expression in *E. coli*^27^. Therefore, 5-ALA was added to the culture medium at the same time as isopropyl-β-D-thiogalactopyranoside (IPTG) induction. As a result, small amount of (*R*)-reticuline was detected in the culture medium of the STORRNcut expression strain, indicating that STORR was functional in *E. coli*, although the N-terminal deletion and 5-ALA addition were required (Supplementary Fig. 3e,f). However, (*R*)-reticuline production by STORR was low (Supplementary Fig. 3f, less than 10 μM) and required the addition of an expensive compound, 5-ALA; therefore, it is not applicable for the practical production of opiates in current conditions. Thus, to construct an opiate production system in *E. coli*, we attempted to develop (*R*,*S*)-reticuline production using two methyltransferases from (*R*,*S*)-THP.

### Functional expression of SalS in *E. coli*

The next step required for the production of opiates is the conversion of (*R*)-reticuline to salutaridine, which is catalysed by the P450 enzyme salutaridine synthase (SalS; Fig. 1, Supplementary Fig. 1)^28^. Human P450s, CYP2D6 and CYP3A4 also have SalS activity, and have been successfully expressed in *E. coli*^27^,^29^,^30^. However, because these P450s produce undesirable by-products^29^, they are unsuitable for the practical production of opiates. Thus, we attempted functional expression of SalS in *E. coli*. We constructed two SalS variants, full-length SalS (SalS) and N-terminus deleted (SalSNcut), as in the case of STORR. These SalS proteins were expressed with ATR2. SDS–PAGE analysis showed that SalSNcut was expressed in *E. coli*, whereas the wild-type SalS was not, suggesting that the N-terminus sequence negatively affected SalS expression in *E. coli*, similar to STORR (Supplementary Figs 2a and 4a). These strains were grown on medium containing pure (*R*,*S*)-reticuline, and we measured the production of salutaridine. According to liquid chromatography mass spectrometry (LC-MS) analysis, the specific peak was observed in the medium of the SalSNcut-expressing strain, and the tandem mass spectrometry (MS/MS) fragment pattern of the peak was almost identical to that of pure salutaridine (Supplementary Fig. 4b,c), indicating that SalSNcut had salutaridine synthetase activity in *E. coli*. In the medium of the full-length SalS-expressing strains, a significant peak was observed at the same retention time as the salutaridine standard (an asterisk in Supplementary Fig. 4b); however, we could not confirm their identity because the MS/MS fragment patterns of the peak was ambiguous. In contrast to STORR, the addition of 5-ALA was not required for salutaridine synthetic activity of SalS (Supplementary Fig. 5), indicating that this property of SalSNcut is adequate for the practical production of opiates. Thus, we decided to use SalSNcut for the total biosynthesis of opiates.

### Thebaine production from authentic (*R*,*S*)-reticuline

**Figure 3:**
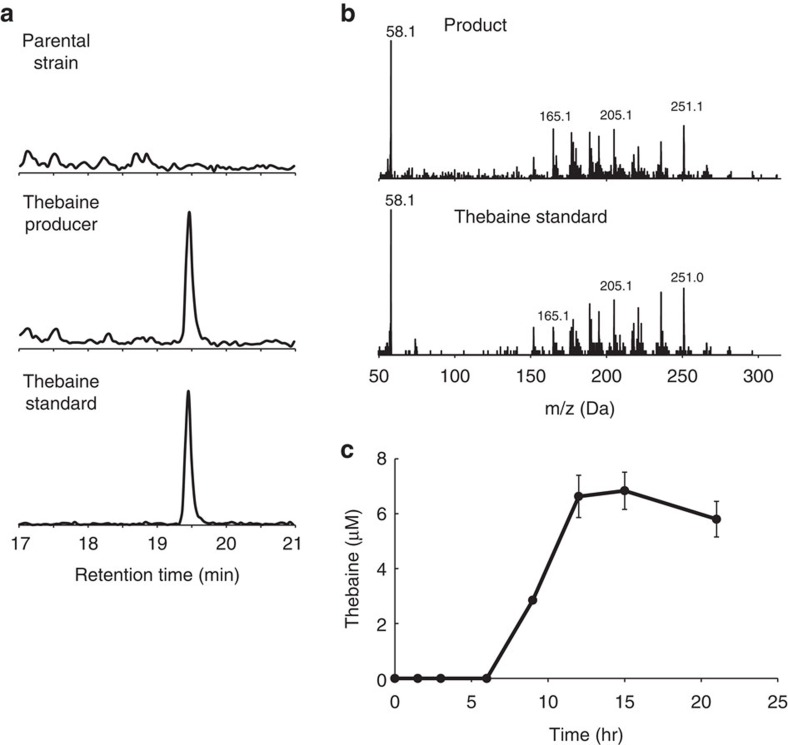
Thebaine production from pure (*R*,*S*)-reticuline or (*R*,*S*)-reticuline synthesized by total biosynthesis. (**a**) LC-MS analysis of thebaine (*m*/*z*=312) from the culture of the parental strain (salutaridine producer, AN1096; upper panel), the thebaine producer (AN1829; mid panel) and the thebaine standard (lower panel). (**b**) MS/MS fragment pattern of the products of AN1829 (upper panel) and the thebaine standard (lower panel). Experiments in **a** and **b** were conducted at least three times, and same tendency was observed. (**c**) Time-course analysis of the total biosynthesis of thebaine from fermentatively produced (*R*,*S*)-reticuline in the four-step culture. (*R*,*S*)-Reticuline synthesized by total biosynthesis was added to the fourth step of the culture at time zero. The error bar indicates the standard deviation of three independent experiments.

To validate the activities of salutaridine reductase (SalR)^31^ and salutaridinol 7-*O*-acetyltransferase (SalAT)^32^, the last two enzymes in the thebaine synthetic pathway (Fig. 1 and Supplementary Fig. 1), their corresponding genes were expressed in the salutaridine-producing *E. coli* strain AN1096 to generate the thebaine-producing strain AN1829. Measurement of thebaine production from authentic (*R*,*S*)-reticuline showed a thebaine-specific peak at *m*/*z*=312 (Fig. 3a). The MS/MS fragment pattern of the peak was almost identical to that of pure thebaine (Fig. 3b), indicating that both SalR and SalAT were functional in *E. coli*. The thebaine yield was 57±4.6 μM using pure (*R*,*S*)-reticuline at 200 μM, containing the R-form at ∼100 μM.

We determined that glucose was essential for thebaine production from (*R*,*S*)-reticuline (Supplementary Fig. 5). In thebaine production, acetyl-CoA is required to convert salutaridinol to salutaridinol 7-*O*-acetate (Supplementary Fig. 1), and acetyl-CoA is synthesized during glycolysis. Furthermore, SalS and SalR require NADPH as a cofactor, which is mainly synthesized in the pentose phosphate pathway during glucose metabolism. These observations explain why glucose is essential for thebaine production.

### Stepwise culture strategy for opiates production

For optimal (*R*)-reticuline production, the amount of (*R*,*S*)-THP should be limited (Fig. 2e). Therefore, the thebaine production pathway should be divided into two: the total biosynthesis of (*R*,*S*)-THP, and thebaine production from (*R*,*S*)-THP. Although tyrosinase is essential for dopamine production in our system (Fig. 1), tyrosinase degrades THP^12^. To avoid the undesirable action of tyrosinase, a stepwise culture method was employed using the dopamine production strain AN1126 and (*R*,*S*)-THP production strain AN1055. Using this method, a total biosynthesis system for (*R*,*S*)-THP was successfully constructed^12^. Thus, for total biosynthesis of thebaine, the thebaine production pathway should be divided into three: dopamine production, conversion of dopamine to (*R*,*S*)-THP and thebaine production from (*R*,*S*)-THP. These pathways were individually constructed in three strains, resulting in the dopamine producer AN1126, a strain that produces (*R*,*S*)-THP from dopamine (AN1055) and AN1998, which produces thebaine from (*R*,*S*)-THP. Although we attempted thebaine production using three-step cultures, thebaine was not produced by the system, regardless of the presence/absence of IPTG. Although IPTG negatively affects (*R*,*S*)-reticuline production (Supplementary Fig. 6), the titre of thebaine was increased by adding IPTG (Supplementary Fig. 5); therefore, we speculated that opposing effects of IPTG on (*R*,*S*)-reticuline and thebaine productions resulted in the low productivity of AN1998 strain. Hence, (*R*,*S*)-reticuline production step would better be separated from the thebaine production step. Thus, we decided to construct the total biosynthesis system using a four-step culture method using four strains: AN1126, AN1055, the (*R*,*S*)-reticuline producer AN1600 and AN1829, which produces thebaine from (*R*,*S*)-reticuline (Fig. 1 and Supplementary Fig. 7).

### Thebaine biosynthesis using a four-step culture system

For the first and second steps, total biosynthesis of (*R*,*S*)-THP was performed as described previously^12^, and the (*R*,*S*)-THP yield was 983 μM (282 mg l^−1^). In the third step, a one-eighth volume of (*R*,*S*)-THP (109 μM) was added to the (*R*,*S*)-reticuline production culture, yielding 16.9 μM (*R*)-reticuline. In the fourth step, the same volume of (*R*,*S*)-reticuline-containing supernatant (final concentration, 8.5 μM) was added to the culture of the thebaine producer. A thebaine yield of 6.8±0.67 μM (2.1±0.21 mg l^−1^) was obtained 15 h post initiation of the fourth step (Fig. 3c). The yields achieved were a 300-fold improvement compared with those of the latest yeast system^8^.

### Hydrocodone biosynthesis using a four-step culture system

**Figure 4:**
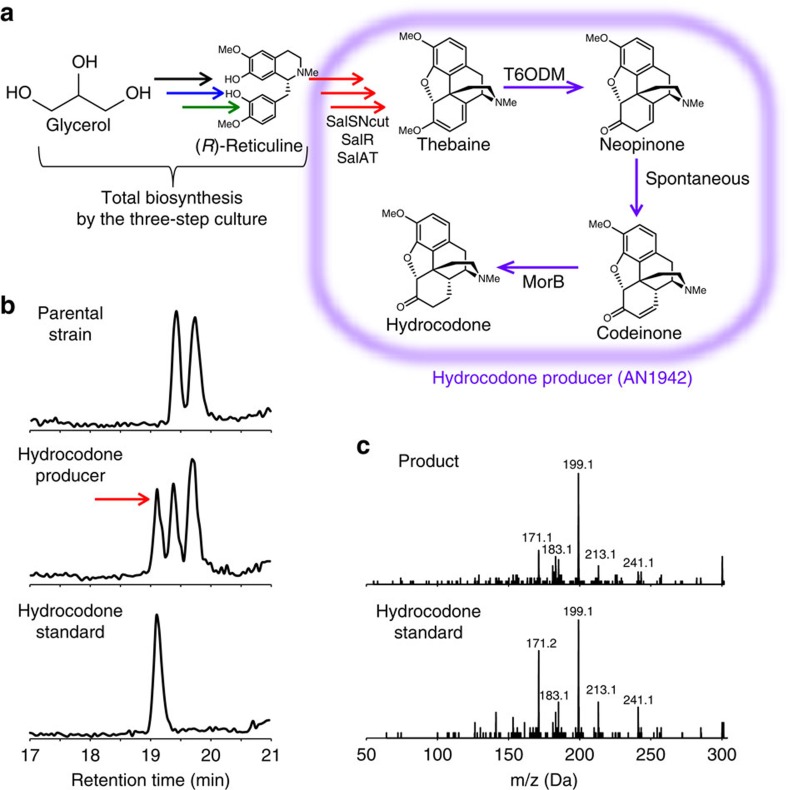
Total biosynthesis of hydrocodone. (**a**) Schematic representation of the total biosynthesis of hydrocodone. (**b**) LC-MS analysis of hydrocodone (*m*/*z*=300) from the culture of the parental strain (thebaine producer, AN1829; upper panel), hydrocodone producer (AN1942; mid panel) and the peak standard of hydrocodone (lower panel). The peak indicated by the arrow was analysed for its MS/MS fragment pattern. (**c**) MS/MS fragment pattern of the products of AN1942 (upper panel) and the peak standard of hydrocodone (lower panel). Experiments in **b**,**c** were conducted three times, and same tendency was observed.

Hydrocodone is one of the most frequently used opioids and can be produced from thebaine using two enzymes, thebaine 6-*O*-demethylase and morphinone reductase (MorB;^33^ Fig. 4a). To evaluate the ability of the thebaine total biosynthesis system to produce medical opioids, we constructed a hydrocodone producer by introducing the genes encoding the two enzymes into the thebaine producer, resulting in strain AN1942 (Fig. 4a). The four-step culture system was used for hydrocodone production. A specific peak in the AN1942 culture was observed, and the MS/MS fragment pattern was identical to that of the hydrocodone standard (Fig. 4b,c). The hydrocodone-producing culture yielded 1.2±0.50 μM (0.36±0.15 mg l^−1^) codeine equivalents of hydrocodone, indicating that the thebaine total biosynthesis system was applicable for the biosynthesis of opioids.

## Discussion

The *E. coli* opiate production system developed in this study does not require specific substrates and resulted in 2.1 mg l^−1^ thebaine production, which is a 300-fold improvement compared with the reported yeast system^8^. This large improvement is associated with high enzyme activity in *E. coli*. In the yeast system, thebaine titres are ∼0.2 μM from 1 mM (*R*,*S*)-THP; the conversion efficiency is typically only 0.02% (ref. 8). However, in the *E. coli* system, the conversion efficiency of thebaine from (*R*,*S*)-THP was 12.5% (6.8 μM thebaine was produced from 54.5 μM (*R*,*S*)-THP diluted via stepwise culture). As the (*R*,*S*)-reticuline production system in yeast exhibits a 10% conversion efficiency of (*R*,*S*)-reticuline from (*R*,*S*)-THP^34^, the large difference in thebaine productivity between *E. coli* and yeast can be attributed to the activity of SalS, SalR and SalAT, which are required for thebaine synthesis from (*R*)-reticuline. In another yeast system, the thebaine conversion efficiency from (*R*)-reticuline is only ∼1% (ref. 7). The conversion efficiency of thebaine from biosynthesized (*R*)-reticuline reached 80% in the *E. coli* system, indicating that the activity of SalS, SalR and SalAT was much stronger in *E. coli* than in yeast. This strong activity may be related to high expression of these enzymes or high productivity of co-factor(s), for example, haeme, NADPH and/or acetyl-CoA in *E. coli*. These results demonstrated that *E. coli* is suitable for opiate production, despite concerns related to the difficulty in expressing plant genes in the species^13^,^34^.

Despite the high conversion efficiency of thebaine from (*R*)-reticuline, the production yield of thebaine was relatively low in this total biosynthesis system. These results suggested that the productivity of (*R*)-reticuline is a major rate-limiting step. To develop a practical production system for opioids, increasing the productivity of (*R*)-reticuline is the greatest challenge. We demonstrated two ways to produce (*R*)-reticuline, that is, using STORR or the irregular activity of a methyltransferase. Although the conversion of racemic THP via methyltransferases was used to reconstruct opiate biosynthesis in this study, half of the reticuline that was produced, (*S*)-reticuline, was not used for opiate production. On the other hand, STORR has low activity in *E. coli*, even after the addition of an expensive cofactor for (*R*)-reticuline production. To improve (*R*)-reticuline productivity, these drawbacks should be overcome by metabolic engineering strategies such as protein engineering for practical production.

Engineered yeast can convert thebaine into various opiates such as morphine^33^. Theoretically, other opioids in addition to hydrocodone can be produced by integrating opioid biosynthetic enzymes such as codeinone reductase (COR), codeine demethylase and morphine dehydrogenase into our thebaine total biosynthesis system. Alternatively, a hybrid *E. coli*-yeast system could enable opioid production^35^. Thus, it is possible, in principle, to produce various opioids from a simple carbon source using microbes.

Stepwise culture has some advantages over single-step culture in the thebaine production system. Because tyrosinase degrades THP, dividing the THP production pathway into two parts enables efficient THP production^12^. Thus, stepwise culture bypasses undesirable reactions in which upstream enzymes act on downstream compounds. The other benefit of stepwise culture is that it permits the individual optimization of each culture step, like chemical synthesis methods. In this study, IPTG had a positive effect on thebaine production, although it diminished (*R*)-reticuline production (Supplementary Figs 5 and 6). Accordingly, dividing the thebaine synthetic pathway from (*R,S*)-THP into two parts via stepwise culture enabled successful opiate production. However, it was not clear why IPTG had the opposite effect. To construct a practical production system, we aim to verify the necessity of separating this step by elucidating the exact mechanism that does not allow the production of thebaine with (*R*,*S*)-reticuline production in our future research.

Recently, microbial production of plant secondary metabolites has attracted attention^5^,^6^,^8^,^13^,^36^,^37^. These compounds require multistep reactions from a simple carbon source, and are associated with undesirable reactions and enzyme inhibition. The efficient production of these compounds may be achieved using stepwise culture, which would minimize problems that arise in multi-step reactions. Furthermore, a stepwise culture method enables the segmentation of a synthetic pathway, and that facilitates the production of similar compounds. In fact, the central intermediate in benzylisoquinoline alkaloid biosynthesis, (*S*)-reticuline, is used for the production of protoberberine with the berberine bridge enzyme, scoulerine *O*-methyltransferase and CYP719A1 (ref. 38). Similarly, aporphine alkaloids are produced from (*S*)-reticuline with CYP80G2 and CNMT enzymes^35^, whereas benzophenanthridine alkaloids are produced via scoulerine with CYP719A5, CYP719A3, TNMT, MSH and P6H^39^. By constructing these enzyme expression strains, and using an (*S*)-reticuline production step, a stepwise culture method can enable the production of various kinds of alkaloids. Thus, stepwise culture methods enable flexibility in the production of various compounds, similar to conventional chemical synthesis methods. The *E. coli* system developed in this study thus represents a potentially useful platform for the further development aimed at the industrial production of opiates.

## Methods

### Plasmids and bacterial strains used in this study

Plasmids and bacterial strains are listed in Supplementary Tables 2 and 3, respectively.

### Construction of pCDF23, pCOLA23 and pAC23

pCDF23, pCOLA23 and pAC23 were generated from pCDFDuet-1, pCOLADuet-1 and pACYCDuet-1, respectively. Each plasmid sequence, other than the T7 promoter to the terminator region containing a multi-cloning site (Pro-MCS-Ter), was amplified by PCR with appropriate primers (Supplementary Table 4). Amplicons were ligated to the Pro-MCS-Ter of pET23a, also obtained by PCR, using an In-Fusion HD cloning kit (Clontech). Thus, the Pro-MCS-Ter regions of pCDFDuet-1, pCOLADuet-1 and pACYCDuet-1 were replaced with Pro-MCS-Ter of pET23a, generating pCDF23, pCOLA23 and pAC23, respectively.

### Gene cloning

All genes were amplified by PCR using the primer sets listed in Supplementary Table 4, and were initially cloned between the *Nde*I and *Bam*HI sites of pET23a, pCDF23 or pAC23 using the In-Fusion HD cloning kit or a conventional ligation method to fuse them with an IPTG-inducible T7 promoter. When required, genes with the T7 promoter were reamplified by PCR using primer set pr339 and pr379, and cloned into the *Xho*I site of the appropriate plasmid. These primers were designed such that only one *Xho*I site at downstream of the cloned gene was regenerated after cloning, meaning that each gene could be cloned in tandem into the vector using the *Xho*I site from one to the next. The plasmids used for total biosynthesis of opiates are represented in Supplementary Fig 5. Codon usage of all genes, except for *tyrA*^*fbr*^, *aroG*^*fbr*^, *tktA*, *ppsA* and *DODC*, was optimized for *E. coli* expression, and these sequences are listed in Supplementary Fig. 8.

### Gene cloning from *Papaver somniferum* L. cv. Ikkanshu

*SalS*, *SalR* and *SalAT* were amplified from a *P. somniferum* L. cv. Ikkanshu cDNA pool using primer sets PsSalSNncS/PsSalSCncA, PsSalRedNncS/PsSalRedCncA and PsSalATNncS/PsSalATCncA, respectively. These fragments were cloned into an *Eco*RV site of vector pT7-Blue (Novagen), and their sequences were analysed (Supplementary Fig. 9).

### Selection of a suitable CPR for expression in *E. coli*

ATR2, ATR2Ncut, PsCPR and RnCPR were expressed in BL21 (AN1067, AN1068, AN1069 and AN1070, respectively). Overnight cultures of these strains were inoculated into 50 ml of TB medium (per litre: 12 g tryptone (Difco), 24 g yeast extract (Diffco), 9.4 g K_2_HPO_4_, 2.2 g KH_2_PO_4_ and 4 ml glycerol) supplemented with 50 mg l^−1^ ampicillin in a baffled shake flask at 25 °C. IPTG (1 mM) was added for induction at 12 h post inoculation. Following further incubation for 12 h, cells were harvested by centrifugation and suspended in 100 mM sodium phosphate buffer (pH 7.3) containing 10% glycerol. The crude extract containing a CPR was obtained by centrifugation following sonication. The protein concentration was measured using a BCA Protein Assay Kit (Pierce). Verification of CPR activity was carried out by a method of P450 reductase activity assay^22^. The reaction mixture contained 2.1 μg of total protein, 100 μg of bovine cytochrome c (Sigma), 100 μM NADPH and 100 mM potassium phosphate (pH 7.3). The samples were incubated at 25 °C for 5 or 10 min. Degradation ratio of NADPH was measured at 550 nm by comparing before with after incubation.

### STORR activity in *E. coli*

Strains expressing full-length STORR or STORRNcut (AN1989 and AN1991, respectively) were cultured in 50 ml of TB medium supplemented with 50 mg l^−1^ ampicillin for 12 h, and then induced by adding 1 mM IPTG with or without 1 mM 5-ALA. Following further incubation for 12 h, biosynthesized (*S*)-reticuline^15^ was added to the cultures (final concentration, 44 μM). The samples were harvested 12 h after the addition of (*S*)-reticuline, and the chirality of reticuline was analysed by LC-MS. For verification of protein expression, the cultures were harvested before (*R*,*S*)-reticuline addition and resuspended in 100 mM sodium phosphate buffer (pH 7.3). The cells were lysed by sonication, and each sample was separated into soluble and insoluble fractions by centrifugation for SDS–PAGE analysis.

### Validating the 6OMT activity of 4′OMT

The crude extract containing the methyltransferase was obtained as described above. (*R*,*S*)-THP, the substrate of the reactions, was synthesized from dopamine following cultivation of strain AN1055, as described previously^12^. The supernatant containing 3.8 mM (*R*,*S*)-THP was applied to an Oasis HLB solid-phase extraction cartridge (Waters), and the eluate was freeze-dried. The standard reaction mixture contained 100 μM (*R*,*S*)-THP, 100 mM potassium phosphate (pH 7.3), 1 mM *S*-adenosylmethionine toluene sulfonate and 22 μg of total protein in 100 μl. The samples were incubated at 37 °C, and the reaction was stopped by adding 2% trichloroacetate. 6-*O*-Methyl THP, 4′-*O*-methyl THP and 6,4′-*O*-dimethyl THP were confirmed by their retention times and MS/MS fragment patterns (daughter ions: 6-*O*-methyl THP, *m*/*z*=178 and 123; 4′-*O*-methyl THP, *m*/*z*=164 and 137; 6,4′-*O*-dimethyl THP, *m*/*z*=178 and 137).

### SalS activity in *E. coli*

Strains AN1096 and AN1420 were cultured in 50 ml of TB medium for 12 h, and then induced by adding 1 mM IPTG. Following further incubation for 12 h, pure (*R*,*S*)-reticuline was added to the cultures (final concentration, 100 μM). The samples were harvested 12 h after (*R*,*S*)-reticuline addition, and the salutaridine content was analysed by LC-MS. SDS–PAGE analysis was conducted as mentioned above.

### Total biosynthesis of (*R*,*S*)-THP using two strains

Total biosynthesis of (*R*,*S*)-THP was performed as previously described^12^. To produce dopamine in the first step, AN1126 was cultured in 1 l of TB medium in a jar-fermenter with glycerol feeding. Dopamine (11.2 mM) was produced from 89 g of glycerol. Dopamine-containing supernatant was harvested by centrifugation and stored at −80 °C until further use. To produce (*R*,*S*)-THP in the second step, AN1055 was cultured in 50 ml of TB medium for 12 h and monoamine oxidase expression was induced by adding IPTG to 1 mM. After further cultivation for 12 h, cells were harvested by centrifugation and resuspended in the same volume of dopamine-containing supernatant. The sample was mixed with 100 mM 2-(*N*-morpholino) ethanesulfonic acid and 10 mM ascorbate and incubated for 12 h at 29 °C. The (*R*,*S*)-THP-containing supernatant was harvested by centrifugation and stored at −80 °C until further use. In this paper, we used two preparations of (*R*,*S*)-THP from total biosynthesis, containing 893 μM (Fig. 2) and 983 μM (in total biosynthesis of opiates) (*R*,*S*)-THP.

### (*R*,*S*)-reticuline production

Strain AN1600 was cultured in 50 ml of TB medium for 24 h. IPTG induction was not performed because IPTG inhibits (*R*,*S*)-reticuline production (Supplementary Fig. 1). The indicated concentration of harvested (*R*,*S*)-THP in Fig. 2e was added to the culture along with 1% glucose, and the culture was incubated for 12 h at 25 °C. For thebaine production, a one-eighth volume of harvested (*R*,*S*)-THP was used. (*R*,*S*)-Reticuline-containing supernatant was harvested by centrifugation and stored at −80 °C.

### Opiate production

Strains AN1829 and AN1942 were cultured in 50 ml of TB medium for 12 h at 25 °C and then induced by adding IPTG to 1 mM. At 12 h post induction, the cultures were mixed with 200 μM pure (*R*,*S*)-reticuline or the same volume of (*R*,*S*)-reticuline synthesized by total biosynthesis, along with 1% glucose. These samples were further incubated at 25 °C.

### Preparation of hydrocodone as a peak standard

Hydrocodone is not commercially available in Japan; therefore, a hydrocodone standard for identification by LC-MS analysis was synthesized from codeine by an enzymatic method using COR and MorB^33^. Crude extracts from COR- and MorB-expressing strains (AN1304 and AN1685, respectively) were obtained by sonication as described above. The hydrocodone synthesis reaction mixture contained 100 mM potassium phosphate buffer (pH 7.3), 1.7 mM codeine, 5 mM NADH and 260 μg ml^−1^ of total protein from the crude extracts. The reaction was conducted at 37 °C for 3 h and stopped by the addition of 2% trichloroacetate. Samples were purified by Oasis HLB solid-phase extraction cartridge (Waters).

### Detection and quantification of chemical compounds

To measure chemical compounds, the culture medium was collected and proteins were precipitated with 2% trichloroacetate. All compounds, except for hydrocodone, were separated by an Agilent HPLC system (Agilent) as follows: column, TSKgel ODS-80Ts (4.6 × 250 mm^2^, 5-μm particles; Tosoh); solvent system, A: 0.1% acetic acid in water, B: 0.1% acetic acid in acetonitrile; gradient modes: 90% A (0–5 min), 90–60% A (5–20 min) and 10% A (20–30 min); flow rate, 0.5 ml min^−1^ at 40 °C. In the case of hydrocodone, samples were separated by TSKgel ODS-100 S (4.6 × 250 mm^2^, 5-μm particles; Tosoh) using solvent A: 0.1% dimethylamine in water and solvent B: 0.1% dimethylamine in acetonitrile, using the same gradient methods described for the other compounds. The chirality of (*R*,*S*)-reticuline was analysed using CHIRALCEL OD-H (4.6 × 250 mm^2^, 5-μm particles; Daicel Chemical Industries) with hexane:2-propanol:diethylamine (72:28:0.1) as a solvent flowing at 0.55 ml min^−1^ at 40 °C. The separated supernatant was analysed by LC-MS and LC-MS/MS (3200 QTRAP, Applied Biosystems) using the selected ion mode: THP (*m*/*z*=288), reticuline (*m*/*z*=330), salutaridine (*m*/*z*=328), thebaine (*m*/*z*=312), codeine (*m*/*z*=300), hydrocodone (*m*/*z*=300) and oxycodone (*m*/*z*=316). Compounds were identified by comparison with pure compounds with regard to their retention time and the fragmentation spectrum in LC-MS/MS. The amounts of THP, reticuline and thebaine were estimated from standard curves using Analyst 1.4.1 software (Applied Biosystems). Hydrocodone concentrations were estimated as equivalents of codeine.

## Additional information

**How to cite this article:** Nakagawa, A. *et al*. Total biosynthesis of opiates by stepwise fermentation using engineered *Escherichia coli*. *Nat. Commun.* 7:10390 doi: 10.1038/ncomms10390 (2016).

## Supplementary Material

Supplementary Information Supplementary Figures 1-9, Supplementary Tables 1-4, Supplementary Note 1 and Supplementary References
