# Supplementary material for: Total biosynthesis of opiates by stepwise fermentation using engineered Escherichia coli
Source: Nat Commun. 2016 Feb 5;7:10390. doi: 10.1038/ncomms10390 (PMC4748248; doi:10.1038/ncomms10390)
Supplement: Supplementary Information — Supplementary Figures 1-9, Supplementary Tables 1-4, Supplementary Note 1 and Supplementary References [file ncomms10390-s1.pdf]

Supplementary Figures

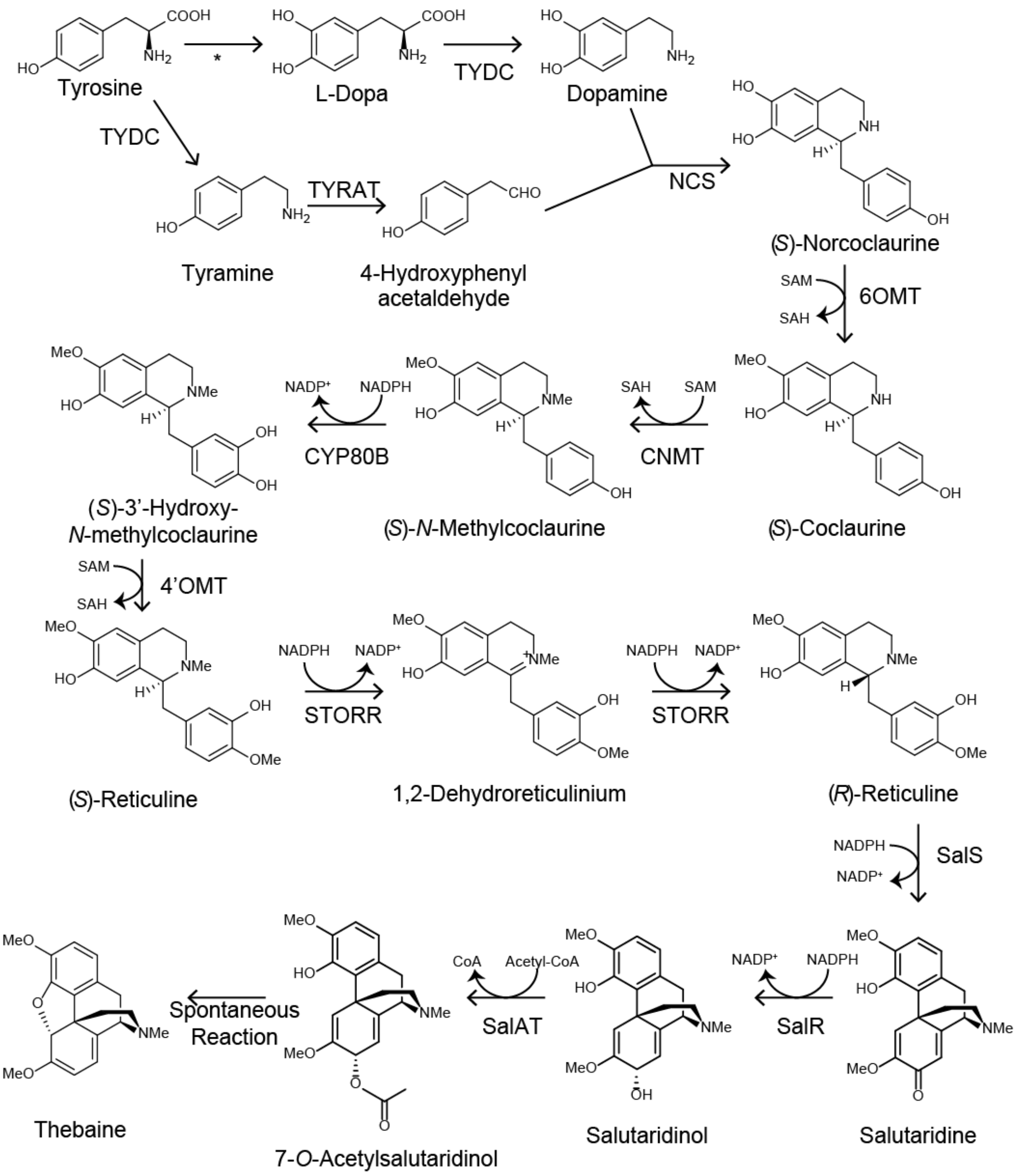

Supplementary Figure 1: Thebaine synthetic pathway in plants. The asterisk indicates an unknown enzyme. 4'OMT, 3'-hydroxy-*N*-methylcoclaurine 4'-*O*-methyltransferase; CNMT, coclaurine *N*-methyltrasferase; 6OMT, norcoclaurine 6-*O*-methyltransferase; CYP80B, *N*-methylcoclaurine 3'-hydroxylase; NCS, norcoclaurine synthase; SalAT, salutaridinol 7-*O*-acetyltransferase; SalR, salutaridine reductase; SalS, salutaridine synthase; STORR, epimerase of (*S*)- to (*R*)-reticuline; TH, tyrosine hydroxylase; TYDC, tyrosine/dopa decarboxylase; TYRAT, tyrosine aminotransferase.

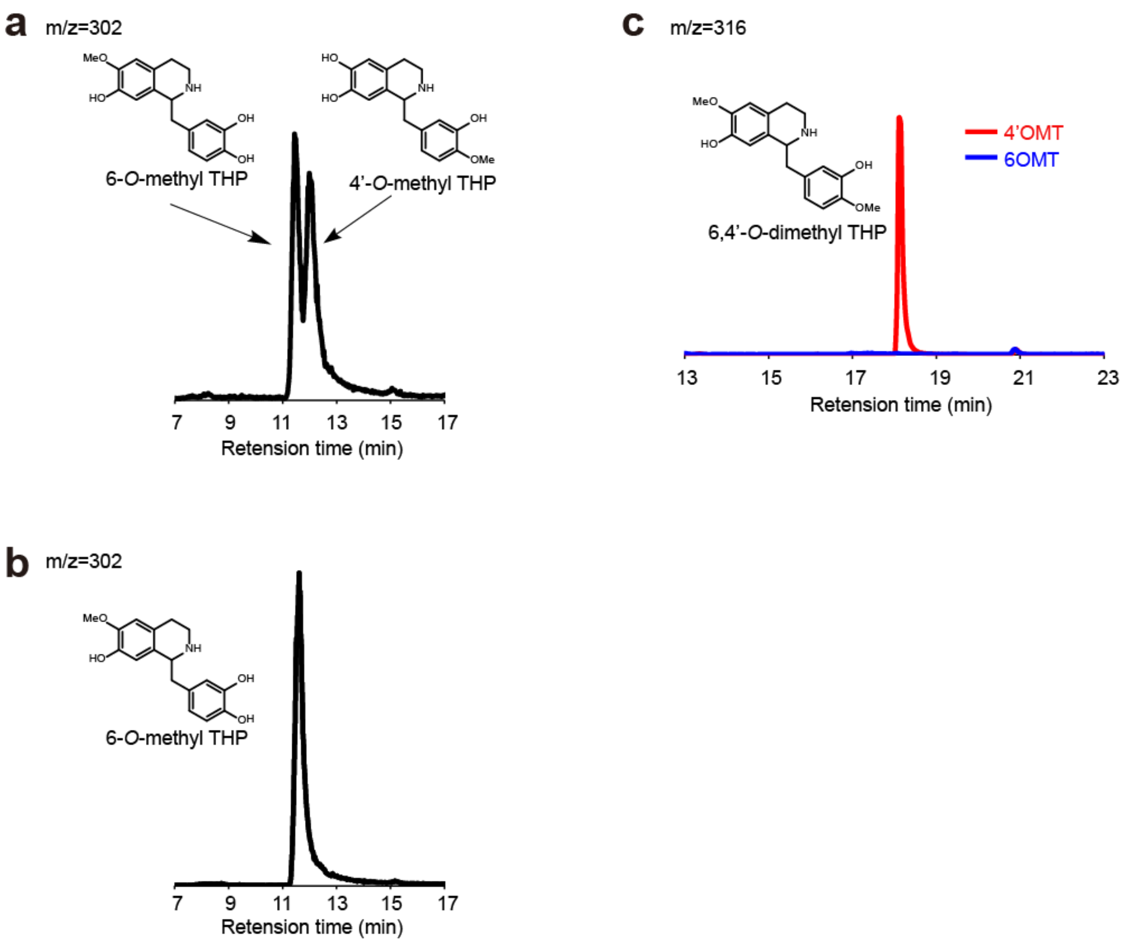

Supplementary Figure 2: **6OMT activity of 4'OMT.** LC-MS analysis of monomethyl-THP (m/z=302) in 4'OMT (**a**) or 6OMT (**b**) reactions. **c**, LC-MS analysis of dimethyl-THP (m/z=316) in 4'OMT (red) and 6OMT (blue) reactions. Experiments were conducted at least three times, and the same tendency was observed.

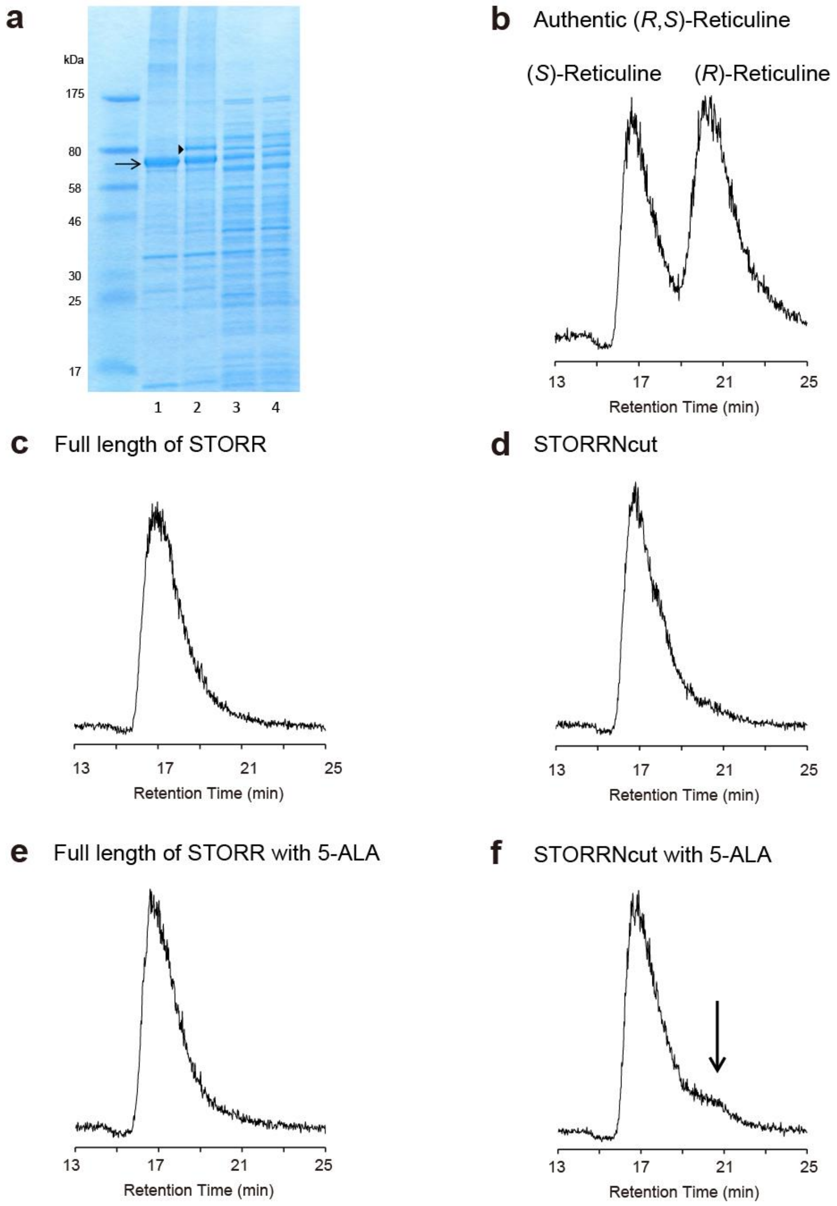

Supplementary Figure 3: STORR activity in *E. coli*. a, Sodium dodecyl sulfate polyacrylamide gel electrophoresis (SDS-PAGE) analysis of STORR and STORRNcut. Lanes 1 and 2: insoluble fraction of sonicated samples; lanes 3 and 4: soluble fraction of sonicated samples; lanes 1 and 3: full-length STORR; lanes 2 and 4: STORRNcut. Arrow: ATR2, triangle: expressed STORRNcut. The chirality analysis of pure (R,S)-reticuline (**b**), the products from the culture of AN1989 without 5-ALA (**c**), the products from the culture of AN1991 without 5-ALA (**d**), the products from the culture of AN1989 with 5-ALA (**e**), and the products from the culture of AN1991 with 5-ALA (**f**). The arrow in **f** indicates the R-form of reticuline. Experiments were conducted three times, and the same tendency was observed.

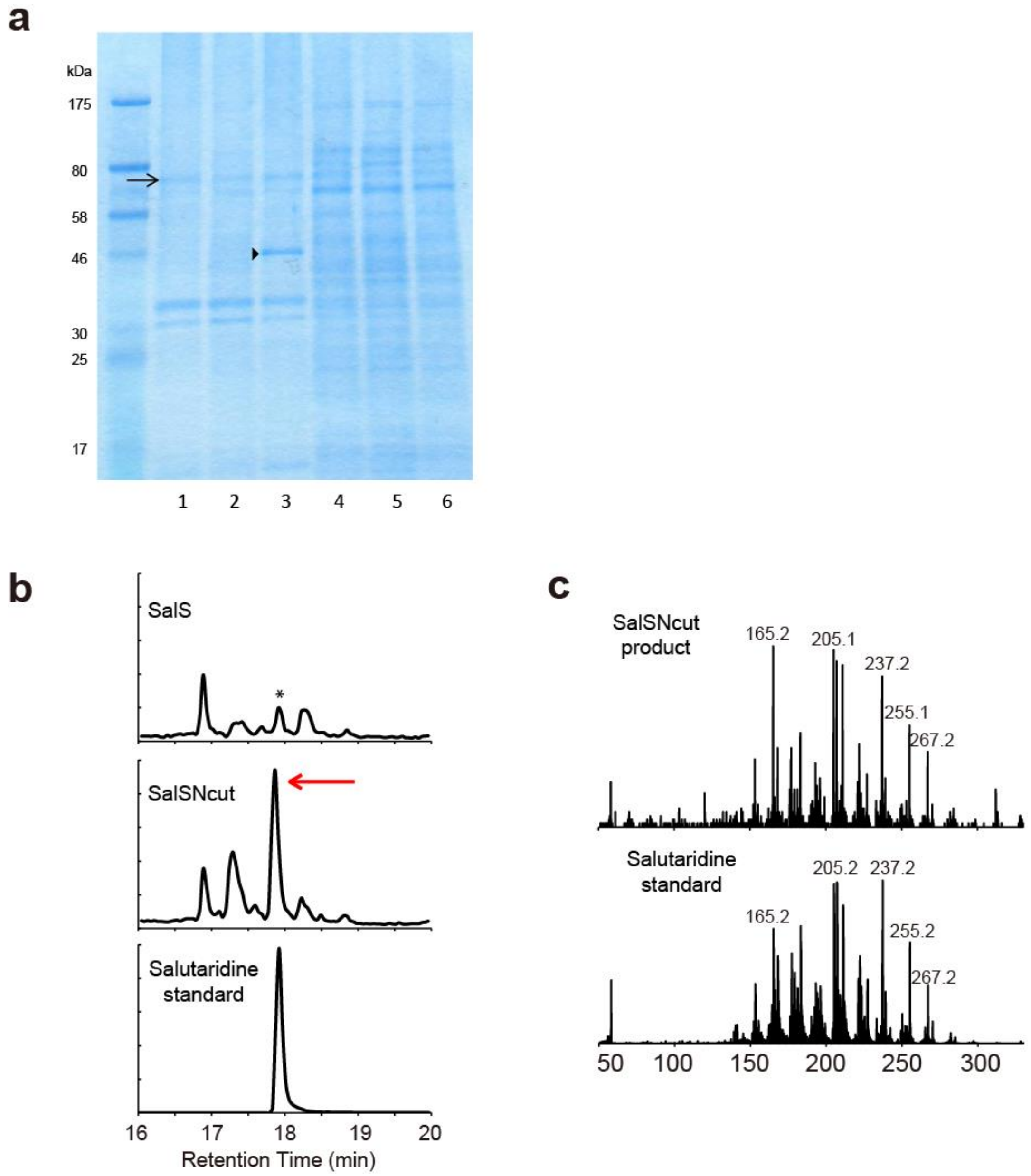

Supplementary Figure 4: SalS expression and productivity of the salutaridine reaction. a, SDS-PAGE analysis of four SalS constructs expressed with ATR2. Lanes 1 and 4: empty vector; lanes 2 and 5: SalS; lanes 3 and 6: SalSNcut. Lanes 1–3: insoluble fractions of sonicated samples; lanes 4–6: soluble fractions of sonicated samples. Arrow: ATR2, triangle: expressed SalSNcut. **b**, LC-MS analysis of the salutaridine content in the culture of each SalS-expressing strain. Asterisk: ambiguous peaks. The peak indicated by arrows was analysed for their MS/MS fragment pattern in **c**. **c**, MS/MS fragment pattern of the salutaridine standard (lower panel), and the products from the culture of SalSNcut-expressing strain (upper panel). Experiments were conducted three times, and the same tendency was observed.

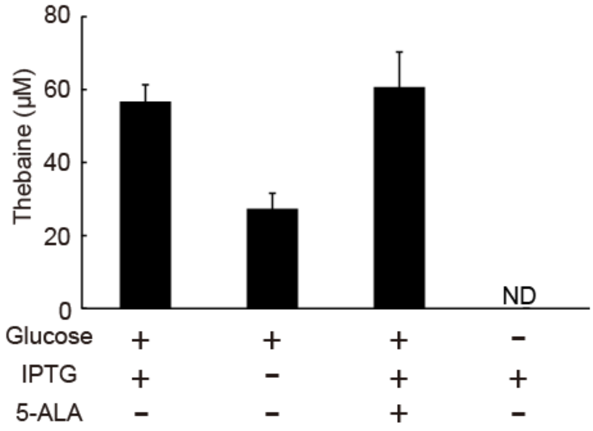

Supplementary Figure 5: Effects of glucose, IPTG and 5-ALA on thebaine production from authentic (*R*,*S*)-reticuline. ND, not detectable. The error bar indicates the standard deviation of three independent experiments.

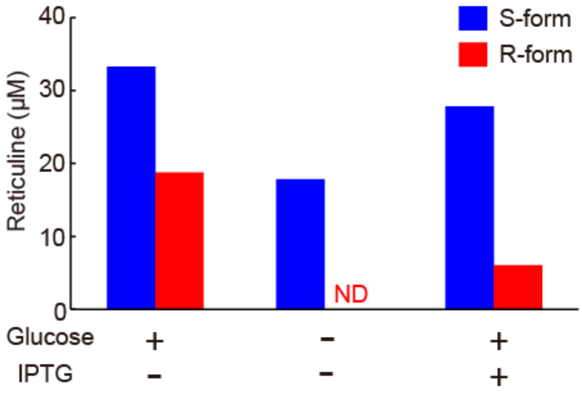

Supplementary Figure 6: Effects of glucose and IPTG on (*R*,*S*)-reticuline production during the third step culture. ND, not detectable. Experiments were conducted at least three times, and the same tendency was observed.

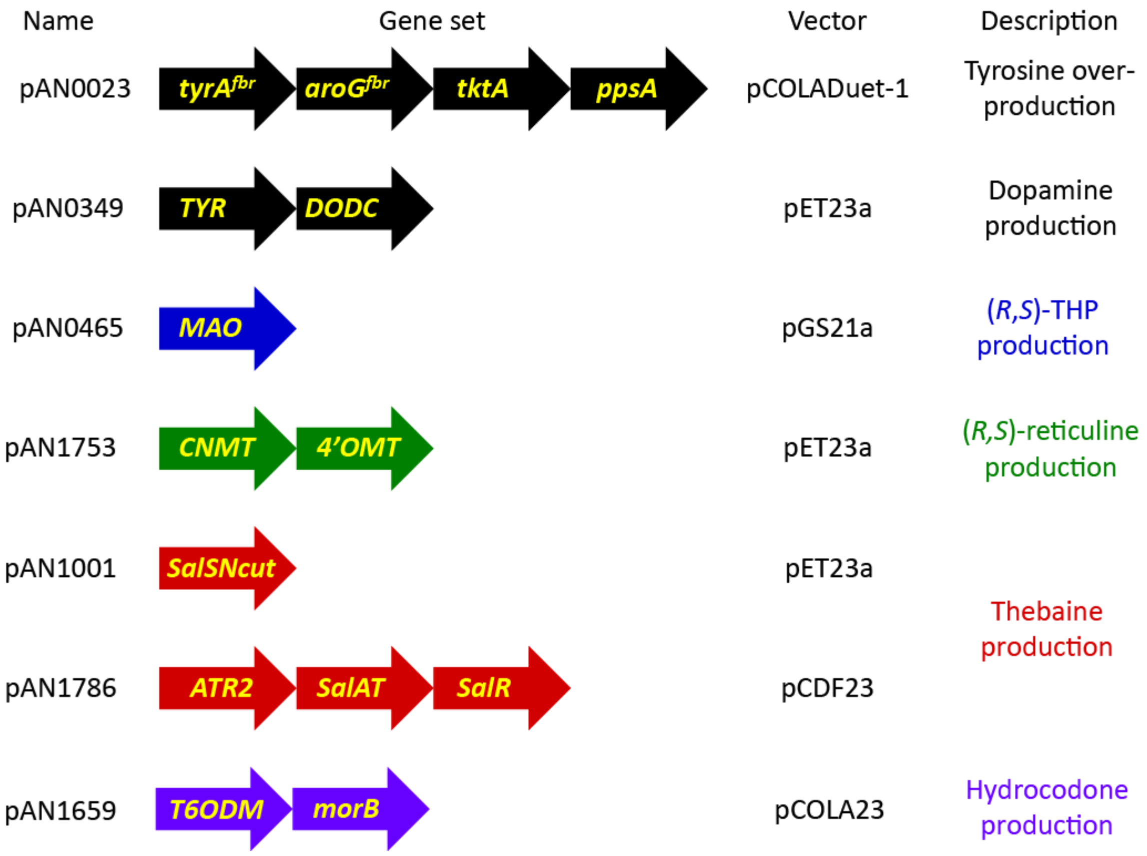

Supplementary Figure 7: Plasmids construction for opioids production. The colors correspond to those in **Figs. 1** and **4**.

4'OMT (3'-hydroxy-*N*-methylcoclaurine 4'-*O*-methyltransferase of *Coptis japonica*; UniProtKB: Q9LEL5).

ATGGCATTCCACGGCAAAGACGACGTTCTGGACATCAAAGCACAGGCACACGTTT
GGAAAATCATCTATGGCTTCGCTGACTCGCTGGTGCTGCGTTGCGCGGTTGAACTG
GGTATTGTCGATATTATCGACAACAATAATCAGCCGATGGCCCTGGCCGATCTGGC
AAGTAAACTGCCGGTTTCCGATGTCAATTGTGACAACCTGTATCGTATTCTGCGCTA
CCTGGTCAAAATGGAAATCCTGCGCGTGGAAAAAAGCGATGACGGTCAGAAAAAA
TATGCGCTGGAACCGATTGCCACCCTGCTGTCACGTAATGCCAAACGCTCGATGGT
GCCGATGATCCTGGGCATGACCCAAAAAGATTTTATGACGCCGTGGCATAGCATGA
AAGATGGTCTGTCTGACAATGGCACCGCGTTCGAAAAAGCCATGGGTATGACGATC
TGGGAATACCTGGAAGGCCACCCGGATCAGAGCCAACTGTTTAACGAGGGTATGG
CAGGCGAAACCCGTCTGCTGACGAGCTCTCTGATTTCAGGTTCGCGCGATATGTTC
CAGGGCATCGATAGTCTGGTGGACGTTGGCGGTGGCAACGGTACCACGGTTAAAG
CAATTTCCGATGCTTTTCCGCATATCAAATGCACCCTGTTCGACCTGCCGCACGTGA
TTGCTAATTCTTATGATCTGCCGAACATTGAACGTATCGGTGGCGACATGTTTAAAA
GCGTTCCGTCTGCACAGGCTATTATCCTGAAACTGATCCTGCATGATTGGAACGAT
GAAGACTCAATCAAAATCCTGAAACAATGTCGCAACGCAGTTCCGAAAGATGGTGG
CAAAGTCATTATCGTCGATGTGGCTCTGGACGAAGAATCGGATCACGAACTGAGTT
CCACCCGTCTGATTCTGGATATCGACATGCTGGTGAATACCGGTGGCAAAGAACGC
ACGAAAGAAGTGTGGGAAAAAATTGTTAAAAGCGCGGGCTTCTCTGGCTGTAAAAT
CCGTCACATCGCCGCTATTCAGTCCGTCATCGAAGTGTTTCCGTAA

6OMT (Norcoclaurine 6-*O*-methyltransferase of *Coptis japonica*; UniProtKB: Q9LEL6)

ATGGAAGTCAAAAAAGACAACCTGTCATCCCAAGCGAAACTGTGGAACTTTATCTA
CGGCTTTGCAGAATCACTGGTGCTGAAATGCGCAGTGCAGCTGGATCTGGCTAACA
TTATCCATAATAGTGGCACCTCCATGACGCTGTCAGAACTGAGCTCTCGTCTGCCG
TCGCAACCGGTGAACGAAGATGCGCTGTATCGTGTTATGCGCTACCTGGTCCACAT
GAAACTGTTTACCAAAGCCAGCATTGATGGCGAACTGCGCTATGGTCTGGCACCGC
CGGCTAAATACCTGGTCAAAGGCTGGGACAAATGCATGGTTGGTTCTATTCTGGCG
ATCACCGATAAAGACTTTATGGCCCCGTGGCATTATCTGAAAGATGGTCTGTCAGG
CGAATCGGGTACCGCGTTCGAAAAAGCCCTGGGCACGAACATTTGGGGTTACATG
GCAGAACACCCGGAGAAAAACCAGCTGTTTAATGAAGCGATGGCCAATGATAGTCG
TCTGATCATGTCCGCTCTGGTGAAAGAATGCGGCAACATTTTCAATGGTATCACCA
CCCTGGTGGATGTTGGCGGTGGCACCGGTACCGCCGTTCGTAACATTGCAAATGCT
TTCCCGCATATCAAATGTACCGTGTATGATCTGCCGCACGTTATTGCAGACAGCCCG
GGCTACTCTGAAGTTCATTGCGTCGCTGGTGATATGTTTAAATTCATCCCGAAAGC

GGACGCCATTATGATGAAATGTATCCTGCACGATTGGGATGACAAAGAATGCATTG
AAATCCTGAAACGTTGTAAAGAAGCAGTCCCGGTGAAAGGTGGCAAAGTGATTATC
GTTGACATTGTCCTGAACGTGCAATCAGAACATCCGTATACCAAAATGCGTCTGAC
GCTGGATCTGGACATGATGCTGAATACCGGTGGCAAAGAACGCACGGAAGAAGAA
TGGAAAAAACTGATCCACGATGCCGGTTACAAAGGTCATAAAATCACGCAAATCAC
CGCAGTCCAGAGTGTCATTGAAGCCTACCCGTATTGA

ATR2 (NADPH--cytochrome P450 reductase 2 of *Arabidopsis thaliana*; UniProtKB: Q9SUM3)
ATGAGCAGCAGCAGTAGCTCTTCCACCAGCATGATTGACCTGATGGCAGCCATTAT
CAAAGGCGAACCGGTTATTGTTAGCGACCCGGCGAACGCATCAGCTTATGAATCGG
TGGCGGCCGAACTGAGCTCTATGCTGATTGAAAATCGTCAGTTTGCGATGATTGTC
ACCACGAGTATCGCCGTGCTGATTGGCTGCATCGTTATGCTGGTCTGGCGTCGCAG
CGGCTCTGGTAACTCCAAACGCGTTGAACCGCTGAAACCGCTGGTCATTAAGCCGC
GTGAAGAAGAAATCGATGACGGCCGCAAAAAGGTTACGATTTTCTTTGGTACCCAG
ACGGGCACCGCGGAAGGTTTCGCGAAAGCCCTGGGTGAAGAAGCAAAGGCTCGTT
ATGAAAAAACCCGCTTTAAGATCGTTGATCTGGATGACTATGCAGCTGATGACGAT
GAATACGAAGAAAAACTGAAAAAGGAAGATGTCGCGTTTTTCTTTCTGGCCACGTA
TGGCGATGGTGAACCGACCGACAATGCGGCCCGTTTCTACAAATGGTTTACCGAAG
GCAACGATCGCGGTGAATGGCTGAAAAATCTGAAGTATGGCGTGTTCGGCCTGGG
TAACCGTCAGTACGAACATTTTAATAAAGTGGCAAAGGTGGTTGACGATATTCTGG
TTGAACAGGGTGCGCAACGCCTGGTTCAGGTCGGCCTGGGTGACGATGACCAATG
TATTGAAGATGACTTTACCGCCTGGCGTGAAGCCCTGTGGCCGGAACTGGACACGA
TCCTGCGCGAAGAAGGTGATACCGCCGTGGCAACCCCGTATACCGCAGCTGTCCT
GGAATACCGTGTGAGCATTCATGATTCTGAAGACGCAAAATTCAACGACATCAATAT
GGCTAACGGCAATGGTTATACGGTTTTTGATGCGCAGCACCCGTACAAAGCGAACG
TGGCCGTTAAGCGTGAACTGCATACCCCGGAATCAGACCGCTCGTGCATTCACCTG
GAATTTGATATCGCCGGCTCAGGTCTGACGTATGAAACCGGCGATCATGTCGGCGT
GCTGTGCGACAATCTGTCGGAAACCGTGGATGAAGCCCTGCGCCTGCTGGATATGT
CACCGGACACGTACTTCTCGCTGCACGCCGAAAAAGAAGATGGCACCCCGATTAGT
TCCAGCCTGCCGCCGCCGTTTCCGCCGTGCAACCTGCGTACGGCACTGACCCGCT
ATGCTTGTCTGCTGTCGAGCCCGAAAAAGAGCGCACTGGTGGCTCTGGCCGCACA
TGCATCTGATCCGACCGAAGCTGAACGTCTGAAACACCTGGCGTCACCGGCCGGT
AAAGATGAATACTCGAAGTGGGTCGTGGAAAGCCAGCGTAGCCTGCTGGAAGTTAT
GGCGGAATTCCCGAGCGCCAAACCGCCGCTGGGCGTTTTCTTTGCGGGTGTTGCT
CCGCGTCTGCAACCGCGTTTTTATAGCATTTCTAGTTCCCCGAAAATTGCGGAAAC

GCGTATCCATGTGACCTGCGCCCTGGTTTACGAAAAAATGCCGACGGGCCGCATCC
ACAAGGGTGTCTGCAGTACCTGGATGAAAAACGCCGTGCCGTATGAAAAGTCCGA
AAATTGTTCATCGGCACCGATTTTCGTCCGTCAGAGCAATTTTAAACTGCCGAGTG
ATTCCAAGGTGCCGATTATCATGATTGGTCCGGGTACCGGTCTGGCACCGTTCCGT
GGCTTTCTGCAAGAACGCCTGGCTCTGGTGGAAAGCGGCGTTGAACTGGGTCCGT
CTGTGCTGTTCTTTGGTTGCCGTAACCGTCGCATGGATTTCATTTATGAAGAAGAA
CTGCAGCGTTTTGTTGAATCTGGCGCACTGGCTGAACTGAGTGTCGCGTTTTCCCG
CGAAGGTCCGACCAAAGAATACGTTCAGCATAAAATGATGGATAAGGCGAGTGACA
TTTGGAATATGATCTCCCAAGGCGCCTATCTGTATGTTTGCGGCGACGCAAAGGGT
ATGGCTCGTGATGTTCATCGCAGCCTGCACACGATCGCGCAGGAACAAGGTAGTAT
GGATTCCACCAAAGCGGAAGGCTTTGTGAAAAATCTGCAAACGAGTGGTCGCTATC
TGCGTGATGTCTGGTGA

CNMT (Coclaurine-*N*-methyltransferase of *Coptis. japonica*; UniProtKB: Q948P7)
ATGGCGGTGGAAGCGAAACAGACGAAAAAAGCTGCGATTGTCGAACTGCTGAAAC
AACTGGAACTGGGCCTGGTGCCGTATGATGATATTAAACAACTGATCCGTCGCGAA
CTGGCACGTCGCCTGCAGTGGGGTTATAAACCGACCTACGAAGAACAGATTGCGG
AAATCCAAAACCTGACGCATTCACTGCGCCAAATGAAAATTGCCACCGAAGTGGAA
ACGCTGGATTCGCAGCTGTATGAAATTCCGATCGAATTTCTGAAAATCATGAACGG
TAGCAATCTGAAAGGCTCTTGCTGTTATTTCAAAGAAGATAGCACCACGCTGGACG
AAGCGGAAATTGCCATGCTGGATCTGTACTGCGAACGCGCGCAGATCCAAGATGG
CCAGTCTGTGCTGGACCTGGGCTGTGGTCAAGGCGCACTGACCCTGCACGTTGCT
CAGAAATATAAAAACTGCCGTGTTACCGCCGTCACGAATAGCGTGTCTCAGAAAGA
ATACATTGAAGAAGAAAGCCGTCGCCGTAACCTGCTGAATGTCGAAGTGAAACTGG
CGGATATCACCACGCATGAAATGGCCGAAACCTATGACCGCATTCTGGTTATCGAA
CTGTTTGAACACATGAAAAACTACGAACTGCTGCTGCGTAAAATTTCAGAATGGAT
CTCGAAAGATGGTCTGCTGTTTCTGGAACATATTTGTCACAAAACCTTCGCCTATCA
TTACGAACCGCTGGATGACGATGACTGGTTTACCGAATATGTTTTCCCGGCCGGTA
CGATGATTATCCCGAGTGCTTCCTTTTTCCTGTACTTTCAGGATGACGTCTCAGTGG
TTAATCATTGGACCCTGAGTGGCAAACACTTCTCCCGCACGAACGAAGAATGGCTG
AAACGTCTGGATGCAAATCTGGACGTCATTAAACCGATGTTTGAAACCCTGATGGG
CAACGAAGAAGAAGCGGTGAAACTGATCAATTATTGGCGTGGTTTCTGCCTGTCCG
GCATGGAAATGTTTGGCTACAATAATGGCGAAGAATGGATGGCGTCTCACGTCCTG
TTCAAAAAAAAAATAA

COR (NADPH-dependent codeinone reductase 1.5 of *Papaver somniferum*; UniProtKB:

BQVRJ2)

ATGGAGTCAAATGGCGTGCCCATGATCACCTTGAGCAGCGGCATTCGCATGCCTGC
TCTGGGGATGGGTACTGTCGAAACGATGGAGAAAGGCACAGAACGCGAGAAACTC
GCCTTTCTGAAAGCGATTGAAGTGGGGTATCGTCACTTTGATACCGCCGCGGCGTA
TCAGACGGAAGAATGTCTGGGTGAAGCCATTGCAGAAGCTCTGCAACTGGGCCTG
ATCAAATCTCGCGATGAACTGTTCATCACGTCCAAACTGTGGTGTGCTGATGCGCA
TGCGGATCTTGTTCTGCCGGCGTTGCAGAACTCGTTGCGCAATCTCAAACTGGATT
ATCTGGATCTGTACCTCATCCACCATCCGGTTAGTCTGAAACCAGGCAAATTCGTG
AATGAAATCCCGAAAGACCACATTCTGCCGATGGACTACAAGAGCGTTTGGGCAGC
TATGGAAGAGTGTCAAACGCTGGGCTTTACCCGTGCAATTGGCGTGTGCAACTTTT
CCTGCAAGAAACTGCAGGAACTCATGGCCACCGCCAATTCGCCACCTGTGGTCAAT
CAGGTGGAAATGAGTCCCACCTTGCATCAGAAGAACTTACGCGAATACTGCAAAGC
CAACAACATCATGATTACCGCGCATAGTGTCTTAGGAGCGGTTGGTGCTGCGTGGG
GTACTAAAGCGGTTATGCATAGCAAGGTACTGCACCAAATTGCGGTCGCACGTGGT
AAATCGGTCGCCCAGGTATCTATGCGGTGGGTATATCAGCAGGGTGCCTCTTTAGT
GGTGAAAAGCTTCAACGAGGCACGCATGAAAGAAAACCTGAAAATTTTCGACTGG
GAACTTACTGCCGAAGATATGGAAAAGATTTCCGAGATCCCGCAATCACGTACCTC
ATCGGCAGCATTCCTTTTAAGCCCGACAGGACCGTTTAAAACGGAAGAGGAATTTT
GGGATGAGAAAGACTAA

MAO (Monoamine oxidase of *Micrococcus luteus*; UniProtKB: C5CB11)

ATGAGCAACCCGCATGTGGTGATTGTGGGTGCAGGTTTTGCAGGCCTGGTGGCGG
CGCGTGAACTGCAGATGGCCGGTGTGGATGTGGAAATTGTGGAAGCGCGTGATCG
TGTGGGCGGCCGTGCATGGACCGAAGAACGTATGGGTCGTCCGCTGGAACTGGGT
GCAACCTGGGTGCATTGGATGCAGCCGCATGTGTGGAGCGAAATTACCCGTTATGA
TCAGAGCATTTATCCGAGCCCGTTTTGCGATGATGCGTATTGGATTACCGGCGGCC
GTGTGGAACATGGTACCGAAGCAGATCTGGATGCAGCACTGGCACGTCCGATGGC
GAAAATTTTTGAAGATAGCCGTGAATTTTTCCCGTATCCGTATGAACCGCTGCATGT
GCTGGATGAAAGCAGCGGCAGCACCCCGGAACTGCGTGAACGTTTTCGTGCGGCG
GATCAGGGCAGCGTGCTGGATTGCCTGAAAGGCGGCGATTTTACCCAGGAAGAAC
GTGATCTGTGCGATGCGTATTGGAGCGCGGCGTATATTGGCGATCCGCATCAGGGC
AGCCCGCTGATGGCGAAACAGTGGGCGGCGCTGAGCGATCATCGTCTGAGCCTGG
TGGATGAACAGACCCTGCGTTTTAAACTGACCCATGGCATGCGTGGCCTGTATGAA
AACATTGCGGCGGATCTGCGTTGCCCGATTCGTCTGAACACCCCGGTGACCGCAG
TGGATCATCGTAGCGATGGTGCAACCGTGACCCTGGGTACCGGTGAAAAAATTAGC
TGCGATAGCGTGATTGTGACCGTGCCGGTGGGTGCACTGCCGACCATTGAATTTAC

CCCGGGTCTGCCGAGCGGTATGCGTACCGTGATTGATCAGCGTTGGAACAGCACC
GGCTGCAAAATTTGGGTGAAAGTGAAAGGCCATCATAGCATTCTGGGCTATGCGCC
GACCCCGCATAAAGCGGCGGTGTTTCGTAGCGAATTTTTCATGGATGATGATACCA
CCATTTGCGTGGGCTTTGGCAGCCATCATGATGCGGTGGATCTGACCGATCCGCGT
GATGCGCAGGCGATTGTGGATCAGTGGCGTCCGGATCTGGAAGTGGTGGATTGCA
CCGGTCATGATTGGGTGGCAGATCGTTGGAGCGGTCAGGCATGGGCAACCCTGCG
TAGCGGCCAGTTTACCAACGGCTGGCATCATTTTCGTAGCACCGATAGCCGTCTGC
GTTTTGCAGGTGCAGATTGGGCGCGTGGCTGGCGTGGCGTGGTGGTGGATGGTGC
AATTGAAACCGGTCTGAGCACCGCGCGTGATGTGCTGCGTGATATTCGTGCGTAA

morB (Morphinone reductase of *Psedomonus putida*; UniProtKB: Q51990)
ATGCCGGATACCAGCTTTTCTAATCCGGGTCTGTTCACGCCGCTGCAGCTGGGTAG
TCTGTCCCTGCCGAATCGTGTGATTATGGCCCCGCTGACCCGTTCACGCACGCCGG
ATAGCGTTCCGGGCCGTCTGCAGCAAATCTATTACGGCCAGCGCGCAAGCGCTGGT
CTGATTATCTCTGAAGCTACCAACATTAGTCCGACCGCGCGCGGCTATGTCTACAC
CCCGGGTATCTGGACGGATGCCCAGGAAGCAGGCTGGAAAGGTGTGGTTGAAGCT
GTGCATGCGAAAGGCGGTCGTATTGCGCTGCAGCTGTGGCATGTGGGCCGCGTTT
CCCACGAACTGGTTCAACCGGATGGTCAGCAACCGGTCGCCCCGTCAGCACTGAA
AGCTGAAGGCGCGGAATGCTTTGTTGAATTCGAAGATGGTACCGCCGGTCTGCAC
CCGACCTCGACGCCGCGTGCCCTGGAAACCGATGAAATTCCGGGCATCGTCGAAG
ACTATCGTCAGGCCGCACAACGTGCAAAACGTGCAGGTTTTGATATGGTTGAAGTC
CACGCAGCTAACGCCTGTCTGCCGAATCAGTTCCTGGCAACCGGTACGAACCGTC
GCACCGATCAATACGGCGGTAGTATTGAAAATCGTGCCCGCTTTCCGCTGGAAGTC
GTGGATGCCGTTGCCGAAGTGTTTGGTCCGGAACGTGTGGGTATCCGCCTGACCC
CGTTTCTGGAACTGTTCGGCCTGACGGATGACGAACCGGAAGCAATGGCTTTTTAT
CTGGCTGGCGAACTGGATCGTCGCGGTCTGGCATACCTGCATTTTAACGAACCGGA
TTGGATTGGCGGTGACATCACCTATCCGGAAGGTTTTCGTGAACAGATGCGTCAAC
GCTTCAAAGGCGGTCTGATTTATTGCGGCAACTATGATGCGGGTCGTGCCCAGGCA
CGTCTGGATGACAATACCGCAGATGCTGTGGCGTTTGGTCGTCCGTTCATTGCCAA
CCCGGACCTGCCGGAACGTTTTCGCCTGGGTGCGGCCCTGAATGAACCGGACCCG
TCTACCTTTTATGGCGGTGCGGAAGTTGGTTATACGGACTACCCGTTCCTGGATAAT
GGCCACGACCGTCTGGGTTAA

PsCPR (NADPH--cytochrome P450 reductase of *Papaver somuniferum*; UniProtKB: O24424)
ATGGTCGACCTCGAGTTAATTAACGTACATATGGGCTCCAACAATCTGGCCAACTCT

ATTGAATCAATGCTGGGTATCAGCATCGGCTCTGAATACATTTCCGATCCGATCTTT
ATCATGGTGACCACCGTGGCTAGCATGCTGATTGGCTTTGGCTTCTTTGCGTGCAT
GAAGTCCAGTAGCTCCCAGAGTAAACCGATTGAAACCTATAAGCCTATCATTGACA
AAGAAGAAGAGGAAATCGAAGTCGATCCGGGTAAAATTAAACTGACTATCTTCTTC
GGCACACAAACCGGTACTGCCGAGGGGTTTGCGAAAGCTTTGGCGGAGGAGATCA
AAGCCAAATACAAGAAAGCAGTCGTAAAAGTCGTGGATCTGGATGATTATGCCGCT
GAGGATGATCAGTACGAAGAGAAGCTGAAAAAAGAATCTCTCGTGTTCTTCATGGT
AGCCACTTATGGTGATGGTGAACCGACCGATAACGCCGCGCGCTTTTACAAATGGT
TTACCCAAGAACATGAACGTGGTGAATGGCTGCAACAGCTGACTTATGGGGTGTTT
GGTTTAGGCAATCGTCAGTATGAGCACTTTAACAAAATTGCGGTAGACGTAGATGA
ACAGCTGGGCAAACAGGGTGCGAAACGCATCGTTCAGGTCGGCCTCGGGGATGAC
GATCAGTGCATTGAGGATGATTTTACGGCATGGCGTGAATTATTGTGGACCGAACT
GGACCAGCTGTTGAAAGACGAAGATGCGGCACCGTCGGTTGCAACGCCGTACATC
GCCACAGTGCCTGAATATCGCGTTGTGATTCACGAAACGACCGTGGCGGCCTTGG
ACGATAAACACATCAATACGGCGAATGGCGATGTTGCATTTGATATCCTGCATCCAT
GCCGGACCATTGTGGCGCAACAGCGTGAACTGCACAAACCGAAAAGCGACCGTTC
TTGCATTCACCTTGAATTTGACATTTCTGGCTCGAGCCTGACTTACGAGACTGGGG
ACCATGTAGGTGTGTATGCTGAGAATTGCGATGAAACCGTCGAGGAAGCGGGAAA
ACTTCTGGGTCAGCCCCTTGACCTCCTGTTTTCAATCCATACGGACAAGGAGGATG
GCAGCCCACAAGGAAGCTCCTTGCCTCCGCCGTTCCCGGGGCCGTGTACGCTGCG
CTCTGCACTGGCTCGCTATGCCGACCTGTTAAACCCGCCACGCAAGGCCAGCTTAA
TCGCCCTGTCTGCGCATGCTAGTGTGCCGAGCGAAGCGGAACGCTTACGCTTCCT
GAGTAGCCCGTTAGGCAAGAACGAATATTCGAAATGGGTGGTCGGATCACAACGCT
CGCTTCTGGAGATTATGGCCGAATTTCCGTCAGCAAAACCGCCCTTGGGTGTGTTC
TTCGCAGCGGTAGCGCCGCGTCTGCCACCGCGCTATTACAGCATTTCGTCCAGTCC
CAAATTTGCCCCGAGCCGTATCCACGTGACGTGTGCACTGGTCTATGGCCAGAGCC
CTACAGGACGTGTTCATCGCGGGGTGTGTTCGACCTGGATGAAACATGCAGTTCCC
CAGGATTCGTGGGCGCCAATTTTCGTTCGCACGAGTAACTTTAAACTGCCTGCTGA
CCCGTCGACACCAATCATTATGGTTGGACCTGGCACCGGTCTGGCTCCCTTTCGTG
GCTTCCTTCAGGAACGCATGGCACTCAAAGAGAATGGCGCGCAACTTGGTCCAGC
TGTCCTGTTCTTTGGTTGCCGTAATCGCAATATGGACTTTATTTATGAAGATGAACT
CAACAACTTCGTGGAACGCGGTGTCATTTCAGAACTGGTTATTGCGTTCAGTCGCG
AAGGCGAAAAGAAAGAATACGTTCAGCATAAAATGATGGAGAAAGCGACCGATGTT
TGGAACGTGATTAGCGGCGATGGCTACCTCTACGTCTGTGGGGATGCGAAAGGCAT
GGCCCGTGATGTACATCGTACCCTGCACACGATTGCCCAAGAACAGGGTCCGATGG
AATCCTCAGCAGCAGAGGCGGCCGTGAAGAAATTACAAGTTGAAGAGCGGTATTT

GCGGGACGTTTGGTAA

RnCPR (NADPH--cytochrome P450 reductase of Rattus novergicus; UniProtKB: P00388)

ATGGGCGATAGCCACGAAGATACCTCAGCGACGATGCCGGAAGCGGTTGCCGAAG
AAGTCTCACTGTTCAGCACGACGGACATGGTCCTGTTTAGCCTGATTGTGGGTGTT
CTGACCTACTGGTTCATCTTCCGTAAGAAAAAGAAGAAATCCCGGAATTCTCTAA
AATCCAGACCACGGCGCCGCCGGTCAAAGAAAGCTCTTTCGTGGAAAAAATGAAG
AAAACCGGCCGCAACATTATCGTGTTTTACGGTAGCCAGACCGGCACGGCAGAAG
AATTCGCTAATCGTCTGAGCAAAGATGCCCATCGTTATGGTATGCGCGGCATGTCT
GCTGACCCGGAAGAATACGACCTGGCGGATCTGAGTTCCCTGCCGGAAATTGATAA
AAGCCTGGTGGTGTTTTGCATGGCTACCTATGGCGAAGGTGACCCGACGGATAACG
CGCAAGACTTCTACGATTGGCTGCAGGAAACCGACGTGGATCTGACGGGTGTGAA
ATTTGCCGTTTTCGGCCTGGGTAACAAAACCTATGAACATTTCAACGCAATGGGTA
AATACGTTGATCAGCGTCTGGAACAACTGGGCGCGCAGCGCATTTTCGAACTGGG
CCTGGGTGATGACGATGGCAATCTGGAAGAAGATTTTATCACCTGGCGCGAACAAT
TCTGGCCGGCCGTTTGTGAATTTTTCGGTGTCGAAGCAACGGGCGAAGAATCATCG
ATTCGCCAGTATGAACTGGTCGTGCACGAAGACATGGATGTCGCGAAAGTGTATAC
CGGTGAAATGGGCCGTCTGAAAAGCTACGAAAACCAAAAACCGCCGTTTGATGCTA
AAAATCCGTTCCTGGCGGCCGTTACCGCGAACCGTAAACTGAATCAGGGTACGGAA
CGCCATCTGATGCACCTGGAACTGGACATTAGCGATTCTAAAATCCGTTATGAAAG
TGGCGATCATGTCGCGGTGTACCCGGCCAACGACTCCGCACTGGTCAATCAGATTG
GTGAAATCCTGGGCGCCGACCTGGATGTTATTATGTCACTGAACAATCTGGATGAA
GAATCGAACAAAAAACACCCGTTTCCGTGCCCGACCACGTATCGCACCGCACTGAC
GTATTACCTGGATATCACCAACCCGCCGCGTACGAATGTGCTGTATGAACTGGCGC
AATACGCCAGTGAACCGTCCGAACAGGAACATCTGCACAAAATGGCGAGCTCTAGT
GGCGAGGGTAAAGAACTGTATCTGTCATGGGTTGTCGAAGCTCGTCGCCATATTCT
GGCGATCCTGCAAGATTACCCGTCGCTGCGTCCGCCGATTGACCACCTGTGCGAAC
TGCTGCCGCGTCTGCAGGCACGCTATTACTCAATTGCATCCTCATCGAAAGTGCAT
CCGAATTCGGTTCACATCTGTGCAGTTGCTGTCGAATATGAAGCCAAAAGTGGTCG
TGTCAACAAAGGCGTGGCAACCTCCTGGCTGCGCGCTAAAGAACCGGCGGGTGAA
AATGGCGGTCGTGCCCTGGTTCCGATGTTTGTCCGTAAAAGCCAGTTTCGCCTGCC
GTTCAAATCTACCACGCCGGTTATCATGGTCGGCCCGGGTACCGGCATTGCTCCGT
TTATGGGCTTCATCCAAGAACGTGCGTGGCTGCGCGAACAGGGTAAAGAAGTGGG
CGAAACGCTGCTGTATTACGGTTGCCGTCGCAGTGACGAAGATTATCTGTACCGTG
AAGAACTGGCCCGCTTTCATAAAGATGGCGCACTGACCCAGCTGAACGTTGCTTTC

TCCCGCGAACAAGCGCATAAAGTGTATGTTCAGCACCTGCTGAAACGTGATCGCGA
ACACCTGTGGAAACTGATTCATGAAGGCGGTGCGCACATCTATGTGTGTGGTGACG
CCCGTAACATGGCAAAAGATGTGCAAAATACCTTTTACGACATTGTTGCCGAATTC
GGCCCGATGGAACATACGCAGGCAGTTGATTATGTGAAAAAACTGATGACGAAAGG
CCGTTATTCTCTGGATGTCTGGTCCTAA

SalAT (Salutaridinol 7-*O*-acetyltransferase of *Papaver somniferum L. cv. Ikkanshu*)
ATGGCGACCATGTATAGCGCGGCGGTGGAAGTGATCAGCAAAGAAACCATTAAACC
GACCACCCCGACCCCGAGCCAGCTGAAAAATTTTAACCTGAGCCTGTTAGATCAGT
GCTTTCCGCTGTATTACTATGTACCGATTATCCTGTTTTATCCGGCCACGGCGGCGA
ATTCTACGGGCTCTAGCAACCATCACGACGATCTGGATCTGCTGAAAAGCTCTCTG
AGCAAAACCCTGGTTCATTTCTACCCGATGGCAGGCCGTATGATTGATAATATTCTG
GTGGATTGCCACGATCAGGGGATTAATTTTTATAAAGTTAAAATCCGTGGCAAAATG
TGTGATTTCATGAGCCAGCCGGATGTGCCGCTGAGCCAGCTGTTACCAAGCGAAGT
TGTGAGCGCGTCTGTTCCGAAAGAAGCGCTGGTGATTGTGCAGGTTAATATGTTTG
ATTGCGGAGGCACCGCGATTTGCAGCTCTGTGAGCCATAAAATAGCCGATGCGGCG
ACCATGAGCACCTTTATTCATAGCTGGGCGAGCACCACCAAAACCAGCCGTTCTGG
CGGCGCAACCGCGAGCGTTACCGATCAGAAACTGATTCCGAGCTTTGATAGCGCGT
CTCTGTTTCCACCGAGCGAACGTCTGACCAGCCCGTCTGGGATGAGCGAGATTCC
GTTTAGCTCTACCCCTGAAGATACCGAGGATGATAAAACCGTGAGCAAACGCTTCG
TGTTTGATTTTGCGAAAATTACGAGCGTGCGTGAAAAACTGCAGGTTCTCATGCAG
GATAACTATAAAAGCCGCCGTCCAACCCGTGTGGAAGTTGTGACCAGCCTGATTTG
GAAAAGCGTGATGAAAAGCACCCCGGCGGGTTTTCTGCCGGTTGTGGATCATGCG
GTGAACCTGCGTAAGAAAATGGACCCGCCGCTGCAGGATGTGAGCTTCGGTAACC
TGAGCGTGACCGTGAGCGCGTTTCTGCCGGCGACAACCACCACGACTACCAACGC
TGTGAACAAGACCATTAACAGCACCAGCTCTGAAAGCCAAGTGGTGCTGCATGAAC
TGCACGATTTTATCGCTCAGATGCGCAGCGAAATTGATAAAGTGAAAGGTGATAAA
GGCAGCCTGGAGAAAGTGATTCAGAACTTTGCGAGCGGCCATGATGCGAGCATTA
AGAAAATTAACGATGTGGAAGTGATTAACTTTTGGATCTCTAGCTGGTGTCGTATG
GGCCTGTATGAAATCGACTTTGGCTGGGGTAAACCGATTTGGGTGACCGTGGATCC
GAACATTAAACCGAATAAAAATTGCTTCTTTATGAACGATACCAAATGCGGCGAAG
GCATCGAAGTCTGGGCGAGCTTTCTGGAAGATGACATGGCGAAATTTGAACTGCAC
CTGAGCGAAATTCTGGAACTGATTTAA

SalR (Saluteridine reductase of *Papaver somniferum L. cv. Ikkanshu*)
ATGCCGGAAACCTGCCCAAACACCGTGACCAAACGTCGCTGCGCGGTAGTGACCG

GTGGCAACAAAGGTATTGGCTTTGAAATCTGCAAACAGCTGAGCTCTAATGGCATT
ATGGTTGTGCTGACTTGCCGTGATGTGACCAAAGGCCTGGAAGCGGTGGAGAAAC
TGAAAAATAGCAACCATGAAAATGTGGTCTTTCATCAGCTGGATGTGACCGATCCG
GTGACCACCATGTCTAGCCTGGCGGATTTTATCAAAACCCATTTTGGCAAATTAGAT
ATCCTGGTCAATAATGCGGGCGTGGCGGGCTTTAGCGTGGATGCCGATCGTTTTAA
AGCGATGATCAGCGATATTGGCGAAGACAGCGAAGAACTGGTGAAAATCTATGAAA
AACCGGAAGCCCAGGAACTGATGACCGAAACCTATGAACTGGCGGAGGAATGCCT
GACCATTAACTATTATGGTGTGAAATCCGTGACAGAAGTACTGATTCCGCTGTTACA
GCTGAGCGATAGCCCGCGTATTGTGAACGTGAGCTCTAGCACCGGCAGCCTGAAAT
ATGTGAGCAACGAAACCGCGCTGGAAATTCTGGGCGATGCGGACGCGCTGACCGA
AGAGCGTATCGATATGGTTGTGAATATGCTGTTAAAGGATTTCAAAGAAAACCTGAT
CGAAACCAATCGTTGGCCGAGCTTTGGTGCAGCGTATACCACCAGCAAAGCGTGTC
TGAACGCCTATACCCGTGTGTTTGCGAAAAAGATTCCGAAATTTCAGGTTAATTGC
GTGTGTCCGGGTCTGGTGAAAACCGAAATGAATTATGGCATTGGGAATTATACCGC
CGATGAAGGTGCGAAACATGTGGTTCGTATTGCGCTGTTTCCGGATGATGGCCCGA
GCGGCTTTTTCTATGATTGTAGCGAACTGTCTGCGTTTTAA

SalS (Salutaridine synthetase of *Papaver somniferum L. cv. Ikkanshu*)
ATGGCGCCGATCAACATTGAAGGCAACGATTTTTGGATGATTGCGTGCACCGTGAT
TATTGTGTTTGCGCTGGTGAAATTTATGTTCTCGAAAATTAGCTTTTATCAGAGCGC
GAATACCACCGAATGGCCGGCGGGCCCGAAAACCCTGCCGATCATTGGCAACCTG
CATCAGCTGGGTGGTGGCGTGCCGCTGCAGGTGGCACTGGCGAATCTGGCGAAAG
TGTATGGCGGTGCGTTTACCATTTGGATTGGTAGCTGGGTGCCGATGATTGTGATC
AGCGATATTGATAACGCGCGTGAAGTGCTGGTTAACAAAAGCGCGGATTATAGCGC
GCGTGATGTGCCGGACATCCTGAAAATTATTACCGCGAATGGCAAAAACATTGCGG
ATTGCGATAGCGGCCCGTTTTGGCATAACCTGAAAAAAGGCCTGCAAAGCTGTATT
AACCCGAGCAACGTGATGAGCCTGAGCCGTCTGCAGGAAAAAGATATGCAGAACC
TGATTAAAAGCATGCAGGAACGTGCGAGCCAGCATAACGGCATCATTAAACCGCTG
GATCATGCGAAAGAAGCGAGCATGCGTCTGCTGAGCCGTGTGATTTTTGGCCACGA
TTTTAGCAACGAAGATCTGGTGATTGGCGTGAAAGATGCGCTGGATGAAATGGTGC
GCATTAGCGGCCTGGCGAGCCTGGCGGATGCCTTTAAAATTGCGAAATATCTGCCG
TCTCAGCGTAAAAACATTCGCGATATGTATGCCACGCGTGATCGTGTGTATAACCTG
ATTCAGCCGCACATTGTGCCGAACCTGCCGGAAAACAGCTTTCTGCATTTCCTGAC
CAGCCAGGATTATAGCGATGAGATTATTTATAGCATGGTGCTGGAAATTTTTGGTCT
GGGCGTGGATAGCACCGCAGCGACCGCGGTGTGGGCGCTGAGCTTTCTGGTGGGC

GAACAGGAAATTCAGGAAAAACTGTATCGTGAAATTAACAACCGTACCGGTGGCCA
GCGTCCGGTGAAAGTTGTGGATCTGAAAGAACTGCCGTATCTGCAGGCGGTGATG
AAAGAAACCCTGCGTATGAAACCGATTGCGCCGTTAGCGGTGCCGCATGTGGCGG
CGAAAGATACGACCTTTAAAGGCCGTCGCATTGTGAAAGGGACCAAAGTGATGGTC
AACCTGTATGCGATCCATCATGATCCGAACGTGTTCCCAGCGCCGTACAAATTTATG
CCGGAACGTTTTCTGAAAGATGTGAACTCTGATGGCCGTTTTGGCGATATTAACAC
CATGGAAAGCAGCCTGATTCCGTTTGGCGCGGGCATGCGCATCTGCGGCGGTGTT
GAACTGGCGAAACAGATGGTTGCGTTCGCACTGGCGAGCATGGTGAACGAATTTA
AATGGGATTGCGTTAGCGAAGGCAAACTGCCGGATCTGAGCGAAGCGATCAGCTTT
ATTCTGTATATGAAAAACCCGCTGGAAGCGAAAATTACCCCGCGCACCAAACCGTT
TCGTCAGTAA

STORR (Bifunctional protein STORR of *Papaver somniferum*; UniProtKB: P0DKI7)
ATGGAACTGCAATACATCTCCTACTTTCAACCGACCTCGTCTGTGGTGGCACTGCT
GCTGGCTCTGGTGTCTATCCTGTCTAGCGTCGTGGTTCTGCGTAAAACCTTTCTGA
ACAATTATAGCTCTAGTCCGGCATCCTCAACCAAAACGGCTGTGCTGTCCCATCAG
CGCCAGCAATCATGCGCCCTGCCGATTTCGGGTCTGCTGCATATCTTCATGAATAA
AAACGGCCTGATCCACGTTACCCTGGGTAATATGGCAGATAAATACGGCCCGATTT
TTAGCTTCCCGACCGGTTCACACCGTACGCTGGTCGTGTCGAGCTGGGAAATGGT
GAAAGAATGTTTTACCGGCAACAATGACACGGCGTTCTCTAACCGCCCGATTCCGC
TGGCGTTTAAAACCATCTTCTATGCCTGCGGCGGTATTGATAGTTACGGTCTGTCTA
GTGTTCCGTATGGCAAATACTGGCGTGAACTGCGCAAAGTCTGTGTGCATAATCTG
CTGAGCAACCAGCAACTGCTGAAATTTCGTCACCTGATTATCTCGCAGGTGGACAC
CAGCTTCAATAAACTGTATGAACTGTGCAAAAACTCTGAAGATAATCATGGTAACTA
CACCACCACCACCACCACCGCCGCGGGTATGGTTCGTATTGATGACTGGCTGGCG
GAACTGAGTTTTAATGTGATTGGCCGCATCGTTTGTGGTTTCCAGTCTGGCCCGAA
AACCGGTGCCCCGAGTCGTGTGGAACAATTCAAAGAAGCAATCAACGAAGCTTCCT
ATTTCATGTCTACGAGTCCGGTCTCAGACAACGTGCCGATGCTGGGTTGGATTGAT
CAGCTGACCGGCCTGACGCGCAATATGAAACATTGCGGTAAAAAACTGGACCTGGT
TGTCGAATCGATTATCAACGATCACCGTCAGAAACGTCGCTTTAGCCGCACCAAAG
GCGGTGACGAAAAAGATGACGAACAAGATGACTTCATTGATATCTGTCTGAGTATC
ATGGAACAGCCGCAACTGCCGGGCAACAATAACCCGAGCCAGATTCCGATCAAATC
TATTGTGCTGGACATGATCGGCGGTGGCACCGATACCACGAAACTGACCACGATTT
GGACGCTGTCCCTGCTGCTGAATAACCCGCATGTCCTGGACAAAGCGAAACAGGA
AGTGGATGCCCACTTTCGTACCAAACGTCGCTCAACGAATGACGCAGCTGCGGCC

GTGGTTGATTTCGATGACATTCGCAACCTGGTGTACATCCAAGCAATCATCAAAGA
ATCAATGCGTCTGTATCCGGCTAGCCCGGTTGTGGAACGTCTGAGCGGTGAAGATT
GCGTTGTCGGTGGCTTTCACGTTCCGGCAGGCACCCGTCTGTGGGCTAATGTCTG
GAAAATGCAGCGCGATCCGAAAGTGTGGGATGACCCGCTGGTTTTTCGTCCGGAT
CGCTTCCTGTCTGACGAACAGAAAATGGTTGATGTCCGTGGTCAAAACTATGAACT
GCTGCCGTTTGGTGCCGGTCGTCGCGTTTGCCCGGGCGTCTCCTTCTCACTGGATC
TGATGCAGCTGGTGCTGACCCGCCTGATTCTGGAATTTGAAATGAAATCGCCGAGC
GGTAAAGTGGACATGACCGCCACGCCGGGCCTGATGAGCTACAAAGTTATTCCGCT
GGATATCCTGCTGACGCATCGTCGCATCAAACCGTGTGTTCAGTCCGCAGCTTCAG
AACGTGATATGGAATCCTCAGGTGTGCCGGTTATTACCCTGGGTTCCGGCAAAGTC
ATGCCGGTGCTGGGTATGGGCACGTTTGAAAAAGTGGGTAAAGGCTCAGAACGTG
AACGCCTGGCGATTCTGAAAGCCATCGAAGTTGGCTATCGTTACTTCGATACCGCG
GCCGCGTATGAAACGGAAGAAGTCCTGGGTGAAGCCATCGCAGAAGCTCTGCAGC
TGGGCCTGGTGAAAAGCCGCGATGAACTGTTTATTTCGAGCATGCTGTGGTGCACC
GATGCCCATGCGGACCGTGTTCTGCTGGCACTGCAAAATTCGCTGCGCAACCTGAA
ACTGGAATATGTCGATCTGTACATGCTGCCGTTCCCGGCCAGCCTGAAACCGGGTA
AAATTACCATGGATATCCCGGAAGAAGACATTTGCCGTATGGATTATCGCTCTGTGT
GGGCTGCGATGGAAGAATGTCAGAATCTGGGCTTTACCAAAAGTATCGGTGTTTCG
AACTTCAGCTGCAAAAAACTGCAGGAACTGATGGCAACGGCTAATATTCCGCCGGC
GGTTAACCAAGTCGAAATGTCGCCGGCCTTTCAGCAGAAAAAACTGCGCGAATACT
GTAACGCAAATAACATTCTGGTCTCTGCTATCAGTGTGCTGGGTAGCAATGGCACC
CCGTGGGGCAGTAACGCGGTTCTGGGTTCCGAAGTCCTGAAGAAAATTGCGATGG
CCAAGGGTAAATCTGTGGCCCAAGTTAGTATGCGTTGGGTGTATGAACAAGGCGCA
TCCCTGGTGGTTAAATCTTTTAGTGAAGAACGTCTGCGCGAAAATCTGAACATCTT
CGACTGGGAACTGACCAAAGAAGATCATGAAAAAATTGGCGAAATCCCGCAGTGT
CGCATTCTGAGCGCGTACTTTCTGGTTAGCCCGAATGGCCCGTTCAAATCTCAAGA
AGAACTGTGGGACGACGAAGCCTGA

T6ODM (Thebaine 6-*O*-demethylase of *Papaver somniferum*; UniProtKB: D4N500)
ATGGAAAAAGCGAAACTGATGAAACTGGGCAATGGCATGGAAATCCCGTCTGTGC
AAGAACTGGCGAAACTGACCCTGGCTGAAATCCCGTCACGTTATGTTTGCGCAAAC
GAAAATCTGCTGCTGCCGATGGGTGCTTCGGTCATTAACGATCATGAAACCATCCC
GGTGATTGACATCGAAAATCTGCTGAGCCCGGAACCGATTATCGGCAAACTGGAAC
TGGATCGCCTGCATTTTGCGTGTAAAGAATGGGGCTTTTTCCAGGTGGTTAACCAC
GGCGTCGATGCCAGCCTGGTGGACAGTGTTAAATCCGAAATTCAGGGCTTTTTCAA

CCTGTCTATGGATGAAAAAACCAAATACGAACAGGAAGATGGCGACGTGGAAGGC
TTTGGTCAGGGCTTCATTGAAAGTGAAGACCAAACCCTGGATTGGGCAGACATCTT
TATGATGTTCACGCTGCCGCTGCATCTGCGTAAACCGCACCTGTTTAGCAAACTGC
CGGTTCCGCTGCGCGAAACCATCGAAAGTTACAGCTCTGAAATGAAAAAACTGAGC
ATGGTGCTGTTTAACAAAATGGAAAAAGCGCTGCAAGTCCAAGCGGCCGAAATTAA
AGGCATGTCTGAAGTGTTCATCGATGGCACGCAGGCAATGCGTATGAACTATTACC
CGCCGTGCCCGCAACCGAATCTGGCTATTGGTCTGACCTCACATTCGGACTTTGGC
GGTCTGACGATTCTGCTGCAAATCAACGAAGTGGAAGGTCTGCAAATTAAACGCGA
AGGCACCTGGATCAGTGTTAAACCGCTGCCGAACGCGTTCGTCGTGAATGTCGGT
GATATTCTGGAAATCATGACGAATGGCATTTATCATTCCGTGGACCACCGTGCGGTT
GTCAACAGCACCAATGAACGCCTGTCTATCGCCACGTTTCACGATCCGTCACTGGA
ATCGGTTATTGGCCCGATCAGTTCCCTGATTACCCCGGAAACGCCGGCCCTGTTCA
AAAGCGGTTCTACCTACGGCGATCTGGTTGAAGAATGTAAAACCCGTAAACTGGAT
GGCAAATCGTTCCTGGATAGTATGCGTATTTAA

TYR (Tyrosinase of *Ralstonia solanacearum*; UniProtKB: Q8Y2J8)
ATGGTCGTTCGTCGCACGGTTCTGAAAGCAATCGCAGGCACCTCGGTCGCCACGG
TCTTCGCAGGCAAACTGACGGGTCTGTCGGCAGTCGCAGCAGATGCAGCTCCGCT
GCGTGTGCGTCGCAATCTGCATGGTATGAAAATGGATGACCCGGATCTGTCAGCCT
ATCGCGAATTTGTGGGTATTATGAAAGGCAAAGATCAGACGCAAGCGCTGTCGTGG
CTGGGTTTCGCCAACCAGCACGGCACCCTGAATGGCGGTTATAAATACTGCCCGCA
TGGTGATTGGTATTTTCTGCCGTGGCACCGTGGCTTCGTCCTGATGTACGAACGTG
CAGTGGCAGCACTGACCGGTTATAAAACGTTTGCTATGCCGTACTGGAACTGGACG
GAAGATCGTCTGCTGCCGGAAGCATTCACCGCTAAAACGTATAACGGCAAAACCAA
TCCGCTGTACGTGCCGAACCGCAATGAACTGACCGGTCCGTATGCACTGACGGATG
CTATTGTGGGCCAAAAAGAAGTTATGGACAAAATCTACGCCGAAACGAACTTTGAA
GTTTTCGGCACCAGCCGTTCTGTCGATCGTAGCGTGCGTCCGCCGCTGGTTCAGAA
TTCTCTGGACCCGAAATGGGTCCCGATGGGCGGTGGCAACCAAGGTATTCTGGAA
CGTACCCCGCATAATACGGTTCACAACAATATCGGCGCGTTTATGCCGACCGCAGC
TTCTCCGCGCGATCCGGTGTTCATGATGCATCACGGTAATATTGACCGTGTTTGGG
CGACGTGGAACGCCCTGGGTCGCAAAAATAGCACCGATCCGCTGTGGCTGGGCAT
GAAATTTCCGAACAATTATATCGATCCGCAGGGTCGTTATTACACGCAAGGCGTTTC
AGACCTGCTGTCGACCGAAGCGCTGGGCTATCGTTACGATGTCATGCCGCGTGCG
GACAACAAAGTGGTTAACAATGCACGCGCTGAACATCTGCTGGCACTGTTTAAAAC
CGGTGATAGTGTCAAACTGGCTGACCATATTCGTCTGCGCTCCGTGCTGAAAGGCG
AACACCCGGTTGCAACCGCAGTCGAACCGCTGAATAGTGCAGTTCAGTTCGAAGC

TGGTACCGTCACGGGTGCGCTGGGTGCAGATGTGGGTACCGGCAGCACCACGGAA
GTCGTGGCACTGATCAAAAACATCCGTATCCCGTACAACGTTATCTCTATCCGCGTT
TTTGTCAACCTGCCGAACGCGAATCTGGATGTGCCGGAAACCGACCCGCATTTTGT
TACGAGTCTGTCCTTCCTGACCCATGCGGCCGGTCACGATCATCACGCACTGCCGA
GTACGATGGTGAACCTGACCGACACGCTGAAAGCGCTGAATATTCGCGATGACAAC
TTCTCCATCAATCTGGTGGCCGTTCCGCAGCCGGGCGTGGCTGTTGAAAGCAGTG
GCGGTGTGACCCCGGAATCCATTGAAGTTGCGGTTATCTGA

Supplementary Figure 8: *E. coli* codon optimized genes used in this study

***SalS* (DDBJ accession number: LC100140)**

ATGGCTCCGATTAATATAGAGGGGAATGATTTTTGGATGATAGCATGCACTGTCATA
ATAGTATTTGCATTGGTGAAGTTCATGTTTTCCAAAATATCTTTTTATCAATCTGCAA
ATACAACGGAATGGCCAGCAGGTCCAAAAACATTACCCATAATTGGAAATCTTCATC
AGTTGGGAGGAGGTGTGCCCTTACAGGTTGCTTTGGCAAACTTAGCTAAAGTTTAT
GGAGGTGCATTTACAATTTGGATTGGAAGCTGGGTTCCAATGATCGTCATAAGCGA
TATCGATAACGCTCGGGAAGTTCTTGTTAATAAATCTGCTGATTATTCCGCTAGAGA
TGTACCTGATATTCTTAAAATCATCACAGCAAATGGGAAGAATATTGCTGATTGTGA
TTCTGGTCCATTTTGGCATAATTTAAAGAAAGGTCTTCAAAGTTGTATAAATCCATC
AAATGTTATGTCTCTATCTCGTTTACAGGAAAAAGACATGCAAAATCTCATCAAATC
CATGCAAGAAAGAGCGTCACAGCATAATGGAATTATAAAACCTCTTGATCATGCCA
AAGAAGCGTCTATGCGATTGCTGAGTAGAGTTATATTTGGTCACGACTTTTCAAATG
AGGATCTCGTTATTGGTGTGAAAGACGCCCTCGATGAGATGGTACGCATAAGTGGG
TTGGCAAGTTTAGCTGATGCTTTTAAAATTGCTAAATATTTACCAAGCCAGAGAAAA
AATATTCGGGATATGTACGCCACAAGAGACAGAGTATATAATTTGATTCAACCACAT
ATCGTCCCTAATCTTCCTGAAAATTCTTTCTTACATTTTCTTACATCTCAAGATTACA
GTGATGAAATTATTTACTCAATGGTACTTGAAATTTTTGGTTTGGGAGTAGATAGTA
CTGCAGCAACGGCAGTTTGGGCACTCTCCTTTTTAGTCGGCGAGCAGGAAATTCAA
GAAAAACTTTACCGCGAAATCAACAACCGGACGGGTGGGCAAAGACCAGTGAAAG
TTGTAGATTTGAAAGAGCTGCCATATCTACAAGCCGTGATGAAAGAAACATTGAGG
ATGAAACCCATCGCACCACTAGCGGTCCCTCATGTAGCAGCAAAAGATACTACATT
CAAGGGGCGGAGAATCGTTAAAGGTACAAAAGTAATGGTGAATCTGTACGCTATCC
ATCACGACCCTAACGTTTTCCCTGCACCGTATAAATTCATGCCAGAGAGATTCTTAA
AGGATGTTAATAGTGATGGACGTTTTGGTGATATCAACACAATGGAAAGTTCGTTG
ATACCATTTGGTGCTGGTATGAGAATTTGTGGAGGTGTAGAATTAGCCAAGCAGAT
GGTAGCTTTTGCTCTTGCAAGTATGGTCAACGAATTCAAATGGGATTGTGTTTCCG
AGGGGAAATTGCCTGATCTTAGTGAAGCTATTAGCTTCATTCTCTACATGAAAAACC
CACTTGAAGCCAAAATTACTCCTCGTACAAAACCTTTTCGACAGTAG

***SalR* (DDBJ accession number: LC100141)**

ATGCCTGAAACATGTCCAAATACTGTTACAAAGAGGAGGTGTGCAGTTGTTACTGG
CGGAAACAAGGGTATTGGATTTGAGATTTGTAAGCAATTATCTTCTAATGGAATCAT
GGTTGTTTTAACTTGTAGAGATGTAACTAAAGGTCTTGAAGCTGTTGAAAAACTCA
AAAATTCTAATCATGAGAATGTGGTTTTTCATCAACTTGATGTTACGGATCCAGTTA
CTACTATGTCTTCTTTAGCGGATTTCATTAAAACACACTTCGGAAAGCTTGATATCT
TGGTAAACAATGCTGGGGTTGCAGGTTTTTCAGTTGATGCTGATCGTTTCAAGGCA

ATGATAAGTGACATTGGAGAGGATTCAGAGGAGCTCGTGAAGATCTACGAAAAACC
AGAAGCCCAAGAATTAATGACAGAGACATATGAATTAGCAGAAGAATGTCTCACAA
TAAATTACTACGGTGTTAAATCGGTAACCGAAGTTCTAATTCCTTTACTTCAACTATC
CGATTCACCAAGAATTGTCAATGTTTCATCATCCACGGGAAGCCTCAAGTATGTATC
CAATGAAACAGCTCTAGAGATACTTGGAGATGCTGATGCATTAACGGAAGAGAGAA
TTGACATGGTAGTGAATATGCTTCTTAAGGATTTTAAGGAAAATTTGATCGAAACAA
ATCGGTGGCCTAGTTTCGGAGCTGCATACACAACATCAAAAGCATGTTTGAATGCG
TACACAAGGGTGTTCGCAAAGAAAATTCCCAAATTTCAGGTCAATTGTGTTTGTCC
TGGTTTGGTTAAAACAGAAATGAACTACGGCATTGGAAATTACACTGCCGACGAAG
GTGCTAAACATGTAGTCAGAATAGCTCTTTTCCCCGACGATGGACCTTCTGGTTTTT
TCTATGATTGTTCAGAACTATCTGCATTTTGA

SalAT **(DDBJ accession number: LC100142)**
ATGGCAACAATGTATAGTGCTGCTGTTGAAGTGATCTCTAAGGAAACCATTAAACC
CACAACTCCAACCCCATCTCAACTTAAAAACTTCAATCTGTCACTTCTCGATCAATG
TTTTCCTTTATATTATTATGTTCCAATCATTCTTTTCTACCCAGCCACCGCCGCTAAT
AGTACCGGTAGCAGTAACCATCATGATGATCTTGACTTGCTTAAGAGTTCTCTTTCC
AAAACACTAGTTCACTTTTATCCAATGGCTGGTAGGATGATAGACAATATTCTGGTC
GACTGTCATGACCAAGGGATTAACTTTTACAAAGTTAAAATTAGAGGTAAAATGTGT
GACTTCATGTCGCAACCGGATGTGCCACTAAGCCAGCTTCTTCCTTCTGAAGTTGT
TTCCGCGAGTGTCCCTAAGGAAGCACTGGTGATCGTTCAAGTGAACATGTTTGACT
GTGGTGGAACAGCCATTTGCTCGAGTGTATCACATAAGATTGCCGATGCAGCTACA
ATGAGTACGTTCATTCATAGTTGGGCAAGCACCACTAAAACATCTCGTAGTGGGGG
TGCAACTGCTTCCGTTACAGATCAGAAACTGATTCCTTCTTTCGACTCGGCATCTCT
ATTCCCACCTAGTGAACGATTGACATCTCCATCAGGGATGTCAGAGATACCATTTTC
CAGTACCCCAGAGGATACAGAAGATGATAAAACTGTCAGCAAGAGATTTGTGTTCG
ATTTTGCAAAGATAACATCTGTACGTGAAAAGTTGCAAGTATTGATGCAGGATAACT
ACAAAAGCCGCAGGCCAACAAGGGTTGAGGTGGTTACTTCTCTAATATGGAAGTCC
GTGATGAAATCCACTCCAGCCGGTTTTTTACCAGTGGTAGATCATGCCGTGAACCT
TAGAAAGAAAATGGACCCCCCATTACAAGATGTTTCATTCGGAAATCTATCTGTAAC
TGTTTCGGCGTTCTTACCAGCAACAACAACGACAACAACAAATGCGGTCAACAAGA
CAATCAATAGTACGAGTAGTGAATCGCAAGTGGTACTTCATGAGTTACATGATTTTA
TAGCTCAGATGAGGAGTGAAATAGATAAGGTCAAGGGTGATAAAGGTAGCTTGGAG
AAAGTCATTCAAAATTTTGCTTCTGGTCATGATGCTTCAATAAAGAAAATCAATGAT
GTTGAAGTGATAAACTTTTGGATAAGTAGCTGGTGCAGGATGGGGTTATACGAGAT
TGATTTTGGTTGGGGAAAGCCAATTTGGGTAACAGTTGATCCAAATATCAAGCCGA

ACAAGAATTGTTTTTTCATGAATGATACGAAATGTGGTGAAGGAATAGAAGTTTGG
GCGAGCTTTCTTGAGGATGATATGGCTAAGTTCGAGCTTCACCTAAGTGAAATCCT
TGAATTGATTTGA

Supplementary Figure 9: Original sequences of *SalS*, *SalR* and *SalAT* from *Papaver somniferum L. cv. Ikkanshu*

Supplementary Tables

Supplementary Table 1: CPRs activity toward bovine cytochrome c

	Reductase activity toward Cyt c (OD_{550} x 10^{-2} /min)
ATR2	8.2 ± 2.5
ATR2Ncut	6.8 ± 1.8
PsCPR	1.4 ± 0.48
RnCPR	5.8 ± 0.92
Empty vector	0.95 ± 0.35

Supplementary Table 2: Plasmids used in this study

Name	Genotype	Description (reference)
pAN0023	pCOLADuet-1-*tyrA*^{fbr}-*aroG*^{fbr}-*tktA*-*ppsA*	The gene set for L-tyrosine over-production (1)
pAN0349	pET23a-*RsTYR-DODC*	The gene set for conversion of L-tyrosine to dopamine (2)
pAN0465	pGS21a-*MAO*	Monoamine oxidase for conversion of dopamine to (*R*,*S*)-THP supplied from Genscript (2)
pAN0466	pET23a-*CNMT*	*CNMT* was amplified with the primer set 5BglT7-pr102 from pUC57-*CNMT* supplied from Genscript, and cloned into BglII-BamHI sites of pET23 with a ligation method.
pAN0467	pET23a-*4'OMT*	*4'OMT* was amplified with the primer set 5BglT7-pr101 from pUC57-*4'OMT* supplied from Genscript, and cloned into BglII-BamHI sites of pET23 with a ligation method.
pAN0490	pET23a-*6OMT*	*6OMT* was amplified with the primer set 5BglT7-pr100 from pUC57-*6OMT* supplied from Genscript, and cloned into BglII-BamHI sites of pET23 with a ligation method.
pAN0840	pET23a-*SalR*	*SalR* was amplified with the primer set pr342-pr343 from pUC57-*SalR* supplied from Genscript.
pAN1001	pET23a-*SalSNcut*	*SalSNcut* was amplified with the primer set pr224-pr226 from pUC57-*SalSN7* supplied from Genscript.
pAN1058	pCDF23-*ATR2*	*ATR2* was amplified with the primer set pr198-5BglT7 from pUC57-*ATR2* supplied from Genscript, and cloned into NdeI-BamHI sites of pCDF23 with a ligation method.
pAN1060	pCDF23-*ATR2Ncut*	*ATR2Ncut* was amplified with the primer set, pr198-pr239 from pUC57-*ATR2* supplied from Genscript, and cloned into BglII-BamHI sites of pCDF23 with a ligation method.
pAN1062	pCDF23-*RnCPR*	*RnCPR* was digested from pUC57-*RnCPR* supplied from Genscript, and cloned into NdeI-BamHI sites of pCDF23 with a ligation method.
pAN1079	pCDF23-*PsCPR*	*PsCPR* was digested from pUC57-*PsCPR* supplied from Genscript, and cloned into NdeI-BamHI sites of pCDF23 with a ligation method.

pAN1183	pCOLA23-*T6ODM*	*T6ODM* was amplified with the primer set pr301-pr302 from pUC57-*T6ODM* supplied from Genscript.
pAN1255	pCOLA23-*COR*	*COR* was amplified with the primer set pr352-pr353 from pUC57-*COR* supplied from Genscript.
pAN1413	pET23a-*SalS*	*SalS* was amplified with the primer set pr223-pr226 from pUC57-*SalS* supplied from Genscript.
pAN1589	pET23a-*CNMT-4'OMT*	4'OMT was amplified with the primer set pr339-pr379 from pAN0467, and cloned into pAN0466.
pAN1643	pET23a-*CNMT-4'OMT-6OMT*	6OMT was amplified with the primer set pr339-pr379 from pAN0490, and cloned into pAN1589.
pAN1649	pET23a-*SalAT*	*SalAT* was amplified with the primer set pr194-pr335 from pUC57-*SalAT* supplied from Genscript, and cloned into NdeI-BamHI sites of pCDF23 with a ligation method.
pAN1653	pAC23-*morB*	*morB* was amplified with the primer set pr339-pr379 from pUC57-*morB* supplied from Genscript.
pAN1664	pCOLA23-*T6ODM-morB*	*morB* was amplified with the primer set pr339-pr379 from pUC57-*morB* supplied from Genscript. This fragment was cloned into pAN1183.
pAN1786	pCDF23-*ATR2-SalAT-SalR*	*SalAT* and *SalR* were amplified with the primer set pr339-pr379 from the cognate plasmids, pAN0840 and pAN1649, respectively. These fragments were sequentially coloned into pAN1058.
pAN1975	pET23a-*STORR*	*STORR* was amplified with the primer set pr506-pr509 from pUC57-*STORR* supplied from Genscript.
pAN1979	pET23a-*STORRNcut*	*STORRNcut* was amplified with the primer set pr507-pr509 from pUC57-*STORR* supplied from Genscript.
pAN1986	pET23a-*CNMT-4'OMT-SalSNcut*	*SalSNcut* was amplified with the primer set pr339-pr379 from pAN1001, and cloned into pAN1589.

Supplementary Table 3: *E. coli* strains used in this study

	Genotype	Description (reference)
BL21(DE3)	F^- *ompT hsdSB*(rB$^-$, mB$^-$) *gal dcm* (DE3)	Supplied from Novagen
AN1028	BL21(DE3) harboring pAN0467	4'OMT over-expression strain
AN1055	BL21(DE3) harboring pAN0465	(*R,S*)-THP producer (2)
AN1067	BL21(DE3) harboring pAN1058	ATR2 expression strain
AN1068	BL21(DE3) harboring pAN1060	ATR2Ncut expression strain
AN1069	BL21(DE3) harboring pAN1079	PsCPR expression strain
AN1070	BL21(DE3) harboring pAN1062	RnCPR expression strain
AN1096	BL21(DE3) harboring pAN1058 and pAN1001	Salutaridine producer (SalSNcut)
AN1126	BL21(DE3) *tyrR* null harboring pAN0023 and pAN0349	Dopamine producer (2)
AN1304	BL21(DE3) harboring pAN1255	Crude extract of this strain was used for preparation of hydrocodone standard.
AN1420	BL21(DE3) harboring pAN1058 and pAN1413	Salutaridine producer (SalS)
AN1472	BL21(DE3) harboring pAN490	6OMT over-expression strain
AN1600	BL21(DE3) harboring pAN1589	(*R,S*)-reticuline producer

AN1685	BL21(DE3) harboring pAN1653	Crude extract of this strain was used for preparation of hydrocodone standard.
AN1752	BL21(DE3) harboring pAN1643	Three methyltransferases expression strain
AN1829	BL21(DE3) harboring pAN1001 and pAN1786	Thebaine producer
AN1942	AN1829 harboring pAN1664	Hydrocodone producer
AN1989	BL21(DE3) harboring pAN1058 and pAN1975	STORR and ATR2 co-expression strain
AN1990	BL21(DE3) harboring pAN1058 and pAN1979	STORRNcut and ATR2 co-expression strain
AN1998	BL21(DE3) harboring pAN1786 and pAN1986	Thebaine producer in three-step culture

Supplementary Table 4: Primers used in this study

Name	Sequence	Target DNA
5BglT7	CCCAGATCTGATCCCGCGAAATTAATACGA	*6OMT, 4'OMT, CNMT, ATR2*
pr100	ATTGGATCCTTAATATGGATAAGCCTC	*6OMT*
pr101	ATTGGATCCTTATGGAAAAACCTCAAT	*4'OMT*
pr102	GCCGGATCCTTATTTTTTCTTGAACAG	*CNMT*
pr194	ATTCATATGGCGACCATGTATAGC	*SalAT*
pr198	CAAGGATCCTCACCAGACATCACG	*ATR2*
pr198	CAAGGATCCTCACCAGACATCACG	*ATR2Ncut*
pr205	GGGAGAGCGTCGAGATCC	pCDF23, pCOLA23, pAC23
pr206	CCGCTGAGCAATAACTAGC	pCDF23, pCOLA23, pAC23
pr207	TCTCGACGCTCTCCCAGATCTGATCCCGCGAAATTAATACGA	Pro-MCS-Ter of pET23a
pr208	GTTATTGCTCAGCGGTGG	Pro-MCS-Ter of pET23a
pr223	ATACATATGGCGCCGATCAACATTG	*SalS*
pr224	CCCCATATGAAAATTAGCTTTTATCAG	*SalSNcut*
pr226	ACTGGATCCTTACTGACGAAACGGTTTGG	*SalS, SalSNcut*
pr239	CCTCATATGCTGATTGAAAATCG	*ATR2Ncut*
pr301	AAGGAGATATACATATGGAAAAAGCGAAACTGATG	*T6ODM*
pr302	GCTCGAATTCGGATCCTTAAATACGCATACTATCC	*T6ODM*
pr335	GCTCGAATTCGGATCCTTAAATCAGTTCCAGAATTTC	*SalAT*
pr339	GGTGGTGGTGCTCGAGTGCGGCCGCAAGCTTGTCG	Tandem construction
pr342	AAGGAGATATACATATGCCGGAAACCTGCCCAAAC	*SalR*

pr343	GCTCGAATTCGGATCCTTAAAACGCAGACAGTTCG	*SalR*
pr352	AAGGAGATATACATATGGAGTCAAATGGCGTGC	*COR*
pr353	GCTCGAATTCGGATCCTTAGTCTTTCTCATCCC	*COR*
pr379	TGCGGCCGCACTCGACGATCCCGCGAAATTAATACGA	Tandem construction
pr506	AAGGAGATATACATATGGAACTGCAATACATCTCC	*STORR*
pr507	AAGGAGATATACATATGCGTAAAACCTTTCTGAAC	*STORRNcut*
pr509	GCTCGAATTCGGATCCTCAGGCTTCGTCGTCCCACAG	*STORR, STORRNuct*
PsSalATCncA	TCATGATTACGGAACACATGTAG	*PsIKSalAT*
PsSalATNncS	GTATCATCTACCATTATCAATCCTG	*PsIKSalAT*
PsSalRedCncA	TGCTGCACTATACGCTGAATC	*PsIKSalR*
PsSalRedNncS	CTTACGTTGATTTTCATTGCTTGAG	*PsIKSalR*
PsSalSCncA	GATCAAGCATCTTCACCCTTG	*PsIKSalS*
PsSalSNncS	CCCCAATCTTTGCAAACCGTC	*PsIKSalS*

Supplementary note 1

In current study, (*R*)-reticuline could be produced without 6OMT. However, we did not know why the R-form of reticuline was produced by the CNMT and 4'OMT expression strain. We previously demonstrated that (*R*)-reticuline formation by three metyltransferases was inhibited by larger amounts of (*R*,*S*)-THP, suggesting that one or more of the methyltransferases have a preference for the S-form of substrates, presumably in a competitive inhibition manner[1]. 6OMT from *P. somniferum* and *C. japonica* do not have stereospecificity toward norprotosinomenine[3] and norcoclaurine[4], respectively. Moreover, partially purified 6OMT from *Argemone platyceras* has equivalent activity toward (*R*)- and (*S*)-THP[5]. Together with the fact that an S-form preference was still observed in the absence of 6OMT (Fig. 2d), CNMT and/or 4′OMT must prefer S-form substrates. 4′OMT from *Berberis koetineana* is an S-form-specific enzyme[6]; therefore, 4′OMT from *C. japonica*, which was used in this study, might have an S-form preference. In this (*R*,*S*)-reticuline production system, the substrates recognized by CNMT and 4′OMT would differ from the original substrates, 6-*O*-methyl THP and 6-*O*-*N*-dimethyl THP, which were formerly synthesized by 6OMT from THP. Therefore, the degree of S-form preference of CNMT and/or 4′OMT might differ between substrates. Alternatively, the S-form preference might differ between the

4'OMT and 6OMT activities of 4′OMT. Regardless, further investigations are required to resolve this issue.

Supplementary references

1 Nakagawa, A. *et al.* A bacterial platform for fermentative production of plant alkaloids. *Nat Commun* **2**, 326 (2011).

2 Nakagawa, A. *et al.* (*R,S*)-tetrahydropapaveroline production by stepwise fermentation using engineered *Escherichia coli*. *Sci Rep* **4**, 6695 (2014).

3 Ounaroon, A., Decker, G., Schmidt, J., Lottspeich, F. & Kutchan, T. M. (*R,S*)-Reticuline 7-*O*-methyltransferase and (*R,S*)-norcoclaurine 6-*O*-methyltransferase of Papaver somniferum - cDNA cloning and characterization of methyl transfer enzymes of alkaloid biosynthesis in opium poppy. *Plant J* **36**, 808-819 (2003).

4 Sato, F., Tsujita, T., Katagiri, Y., Yoshida, S. & Yamada, Y. Purification and characterization of S-adenosyl-L-methionine: norcoclaurine 6-*O*-methyltransferase from cultured *Coptis japonica* cells. *Eur J Biochem* **225**, 125-131 (1994).

5 Rueffer, M., Nagakura, N. & Zenk, M. H. Partial Purification and Properties of S-Adenosylmethionine: (*R*), (*S*)-Norlaudanosoline-6-*O*-Methyltransferase from *Argemone platyceras* Cell Cultures. *Planta Med* **49**, 131-137 (1983).

6 Frenzel, T. & Zenk, M. H. S-adenosyl-l-methionine: 3'-hydroxy-N-methyl-(*S*)-coclaurine-4'-*O*-methyl transferase, a regio- and stereoselective enzyme of the (*S*)-reticuline pathway. *Phytochemistry* **29**, 3505-3511 (1990).
